# Specific and conserved patterns of microbiota-structuring by maize benzoxazinoids in the field

**DOI:** 10.1186/s40168-021-01049-2

**Published:** 2021-05-07

**Authors:** Selma Cadot, Hang Guan, Moritz Bigalke, Jean-Claude Walser, Georg Jander, Matthias Erb, Marcel G. A. van der Heijden, Klaus Schlaeppi

**Affiliations:** 1 Division of Agroecology and Environment, Agroscope, Zurich, Switzerland; 2 Department of Environmental Sciences, University of Basel, Bernoullistrasse 32, 4056 Basel, Switzerland; 3 Institute of Plant and Microbial Biology, University of Zurich, Zurich, Switzerland; 4 Institute of Geography, University of Bern, Bern, Switzerland; 5 Genetic Diversity Centre, D-USYS, ETH Zurich, Zurich, Switzerland; 6 Boyce Thompson Institute, Ithaca, NY USA; 7 Institute of Plant Sciences, University of Bern, Bern, Switzerland; 8 Institute of Environmental Biology, Utrecht University, Utrecht, The Netherlands

**Keywords:** *Zea mays*, Root exudates, Benzoxazinoids, Rhizosphere, Root microbiota

## Abstract

**Background:**

Plants influence their root and rhizosphere microbial communities through the secretion of root exudates. However, how specific classes of root exudate compounds impact the assembly of root-associated microbiotas is not well understood, especially not under realistic field conditions. Maize roots secrete benzoxazinoids (BXs), a class of indole-derived defense compounds, and thereby impact the assembly of their microbiota. Here, we investigated the broader impacts of BX exudation on root and rhizosphere microbiotas of adult maize plants grown under natural conditions at different field locations in Europe and the USA. We examined the microbiotas of BX-producing and multiple BX-defective lines in two genetic backgrounds across three soils with different properties.

**Results:**

Our analysis showed that BX secretion affected the community composition of the rhizosphere and root microbiota, with the most pronounced effects observed for root fungi. The impact of BX exudation was at least as strong as the genetic background, suggesting that BX exudation is a key trait by which maize structures its associated microbiota. BX-producing plants were not consistently enriching microbial lineages across the three field experiments. However, BX exudation consistently depleted *Flavobacteriaceae* and *Comamonadaceae* and enriched various potential plant pathogenic fungi in the roots across the different environments.

**Conclusions:**

These findings reveal that BXs have a selective impact on root and rhizosphere microbiota composition across different conditions. Taken together, this study identifies the BX pathway as an interesting breeding target to manipulate plant-microbiome interactions.

Video Abstract

**Supplementary Information:**

The online version contains supplementary material available at 10.1186/s40168-021-01049-2.

## Background

Plants accommodate a specific and species-rich microbiota, including bacteria and fungi, on and in their roots, and in the rhizosphere. The rhizosphere refers to the soil zone surrounding the roots that is impacted by plant exudates (see below). Previous studies have shown characteristic microbiotas of root-associated compartments for plant species, including *Arabidopsis thaliana* [1, 2], *Oryza sativa* [3], *Agave spp.* [4], *Populus deltoides* [5] and *Zea mays* [6]. Similar to the microbial communities in human or animal guts, these microbes collectively function as a microbiome and impact host performance [7]. Beneficial traits of the plant root-associated microbes are manifold and include hormone-mediated plant growth promotion [8], contribution to nutrient uptake [9–11], direct protection against pathogens [12], indirect modulation of the plant immune system to enhance pathogen resistance [13], and improving abiotic stress tolerance [14, 15]. The composition of plant root and rhizosphere microbiotas primarily reflects their origin from the surrounding soil microbiota, which is edaphically determined by the physico-chemical properties of a soil [7, 16].

In addition to ‘soil properties’ as a main driver of plant microbiota composition, the plant genotype has a smaller impact, explaining around 5% of the variation in microbiota composition [7, 16]. Plants mainly influence their root and rhizosphere microbial communities through the secretion of root exudates, which probably present the mechanistic link between host genetic variation and observed differences in microbiota composition between different genotypes [17]. It is likely that the combination of root exudates, the microbes’ substrate preferences and their competitiveness for the diverse carbon sources result in the specific composition of a microbiota [18]. Root exudates consist of a diverse array of exuded chemicals, with general compounds such as organic acids or sugars that mainly serve as nutritional carbon sources, and specialized compounds with semiochemical or toxic properties that further sculpt microbiota composition [17, 19]. While the plant genotype, which defines a plant’s exudation profile, is recognized as an important factor for plant microbiota assembly, relatively little is known about specific classes of exudate compounds impacting root and rhizosphere microbiota composition. It is unknown whether there are conserved microbiota responses to root exudation patterns across different soils. The existence of widespread and conserved response patterns presents an important basis for managing microbial communities with exudate chemistry.

Recent work focusing on specialized exudate compounds, for example coumarins or benzoxazinoids (BXs), points to a selective function of such secondary metabolites in plant microbiota assembly. Coumarins are abundant phenylalanine-derived specialized metabolites occurring in many plant families [20]. Recently, coumarins were reported to shape the root-associated microbiota of Arabidopsis when secreted from the roots [21, 22]. Scopoletin, the predominant coumarin, exerts direct antimicrobial activity against soil-borne fungal pathogens but not against growth-promoting and systemic resistance-inducing rhizobacteria, suggesting that plants assemble a health-promoting root microbiota through coumarin exudation [21]. Benzoxazinoids (BXs) are indole-derived specialized compounds of the *Poaceae* including major crops like maize, wheat and rye [23]. Maize secretes substantial amounts of BXs to the rhizosphere, thereby impacting the assembly of the root and rhizosphere microbiota [24–26]. BXs are primarily known for their chemical defense functions against herbivores, pathogens and competing plant species [27]. However, their ecological repertoire is broader [28] as they can also function as defense signaling molecules [29, 30] and phytosiderophores for iron uptake [31].

Similar to coumarins, the secretion of BXs appears to assemble more health-promoting root and rhizosphere microbiotas. We demonstrated recently that root and rhizosphere microbial communities, which were conditioned by exuded BXs, enhanced jasmonate signaling and defenses against insect herbivores in the next plant generation [24]. While these BX-driven plant-soil feedback present an indirect health promoting function of root and rhizosphere microbiotas, BXs also appear to function directly against soil-borne fungal pathogens. Evidence comes from recent studies comparing BX effects on root and rhizosphere microbiotas that found reduced abundances of fungal sequences with taxonomic links to plant pathogens on BX-producing plants [25, 26]. BXs were found to reduce the virulence of the plant pathogen *Agrobacterium tumefaciens* through bacteriostatic effects [32], and attracting resistance-inducing rhizobacterium *Pseudomonas putida* [33, 34]. Taken together, these findings suggest that BXs in maize root exudates function in assembling a more health-promoting root microbiota.

Several genetically different maize lines are available for studying the effects of BX exudation on microbiota composition. The maize genetic backgrounds B73 and W22 primarily accumulate the BXs 2,4-dihydroxy-7-methoxy-1,4-benzoxazin-3-one glucose (DIMBOA-Glc), DIMBOA, and *N*-*O*-methylated DIMBOA-Glc (HDMBOA-Glc [31];). Mutant lines in BX1, the first enzyme of the BX biosynthesis pathway, which converts indole-3-glycerolphosphate to indole, were identified in both genetic backgrounds. *Bx1*(B73) and *bx1*(W22) are both deficient in the accumulation and secretion of BXs [24, 31]. BX2 encodes the second enzyme of the BX biosynthesis that converts indole to indolin-2-one. The knock-out mutant *bx2*(W22) phenocopies the BX deficiency of *bx1* mutants [31]. BX6 is a downstream enzyme in the BX pathway that acts in the multi-step conversion of DIBOA-Glc to DIMBOA-Glc. Consequently, the BX profile of the mutant *bx6*(W22) differs in its speciation from wild-type (WT) plants [31]. Overall, the mutant *bx6*(W22) produces 15% less BXs, in particular lower levels of DIMBOA and its glucoside, and instead accumulates higher amounts of DIBOA-Glc (the precursor of DIMBOA-Glc).

Earlier studies focused on BX-dependent microbial feedbacks [24] were investigating 17-day-young plants [25] or were conducted in semi-artificial rhizobox systems [26] but were not comparing BX impacts on microbiotas in different soils under field conditions. As microbiota assembly can be highly dependent on context and soil properties, we investigated the broader impacts of BX exudation on the root and rhizosphere microbiotas of 3-month-old maize plants grown under agriculturally relevant conditions at different field locations in Europe and the USA. We were interested in the following specific research questions: (i) How do soils, rhizosphere and roots compare in their microbiota responses to BX exudation? (ii) What is the influence of the plant genetic background on BX-mediated microbiota effects? (iii) How do different mutations in the BX biosynthesis pathway shape rhizosphere and root microbial communities? and (iv) Is there a core group of microbial taxa that consistently responds to BX exudation across the different conditions?

We approached these questions by conducting two field experiments, one in a loamy soil in Aurora, NY (USA), and the other in a clay loam soil in Reckenholz near Zurich (Switzerland), where we grew the various mutant lines (*bx1*, *bx2* and *bx6*) in the two genetic backgrounds, B73 and W22. We profiled soil, rhizosphere and root microbial communities utilizing the same method as in our earlier work and compared the data from the two new experiments to the existing microbiota profiles from plants grown in a field in Changins, Switzerland (clay loam soil [24];). In summary, we showed that the BXs affected community composition of rhizosphere and root compartments and we found that BX-producing plants accumulate lower levels of *Flavobacteriaceae* and *Comamonadaceae*, as well has more abundant potential plant pathogenic fungi, as a conserved microbiota response pattern.

## Methods

### Plant genotypes

We examined the *Zea mays* L. mutant line *bx1*(B73), representing a near-isogenic line that was backcrossed five times to its wild-type (WT) genetic background B73 [35]. We also worked with the mutants *bx1*(W22), *bx2*(W22) and *bx6*(W22) and their WT genetic background W22 [36, 37]. The mutants in W22 have slightly different genetic backgrounds, because they were identified in *Ds* transposon lines with different anthocyanin genes (a1 and r1) as transposon launch sites. The mutants *bx1*(W22) and *bx6*(W22) were identified in W22 r1-sc:m3 (also known as T43) whereas *bx2*(W22) originates from W22 a1-m3. We utilized the W22 line with the r1-sc:m3 mutation as reference.

### Field experiments

The experiment in Reckenholz (Switzerland) was conducted in 2016 and consisted of WT and *bx1* mutant lines in both genetic backgrounds (B73 and W22), which we grew as single plants separated by maize hybrids in a field at Agroscope (Parcel 209, 47° 25′ 34.5′′ N 8° 31′ 05.9′′ E). Seed stock leftovers of various maize hybrid varieties were mixed and planted as buffer between the test plants. To avoid having the maize hybrids outgrow the B73 and W22 inbred lines, we pre-grew these plants for three weeks in the greenhouse in 7 × 7 × 9 cm pots, which were filled with 5-mm-sieved field soil from Parcel 209. The field was ploughed (20-cm depth) and harrowed in April 20, and then in May 9 after harrowing a second time, the seeds of the hybrid plants were sown. In May 27, we replaced individual hybrid plants by transplanting the pre-grown inbred line plants (including the soil in their pots) to the field. The field setup was such that we grew 4 rows containing test plants, which were separated by one buffer row of hybrid plants between them. Two rows of hybrid plants surrounded the field site (Figure S1A). The four genotypes B73, *bx1*(B73), W22 and *bx1*(W22) were mixed and spaced within the rows by 1 m with hybrid plants in between them. Field management followed conventional farming practices consisting of herbicide applications (June 27: 1.5 l/ha of Landis (44 g/l Tembotrione and 22 g/l Isoxadifen-ethyl) and 1.5 l/ha Terbuthylazin (333 g/l) and Flufenacet (200 g/l) and mineral fertilizer application, 2.5 dt/ha of magnesium-ammonium nitrate and 1 dt/ha of urea (June 24) and 1 dt/ha of urea (July 6). Preceding crops were winter barley (2015) and potato (2014). Single plants were harvested on August 08, 3 months after sowing, before they started flowering.

The maize lines W22, *bx1*(W22), *bx2*(W22) and *bx6*(W22) were sampled in a field experiment in Aurora (USA, 2016) at the Cornell Musgrave Research Farm in Aurora, NY (Field U, 42.43′ 23′′ 84° N; −76.39′ 28′′ 84° E). The field was chiseled, ploughed to 18 cm and disked for seedbed preparation. Sowing was on May 18. The four genotypes were planted together with many other maize lines for seed bulking, and all maize lines were grown in rows of 5.6 m length (~20 plants per row) per block (Figure S1B). We collected the mutants *bx1*(*W22*), *bx2*(W22) and *bx6*(W22) from two different blocks. Field management was according to conventional farming practices consisting of weed control (herbicide application of 0.6 l/ha Metolachlor and 0.9 l/ha Atrazine), and fertilization with 0.9 dt/ha 10-20-20 NPK at planting and 0.54 dt/ha N in the form of urea and ammonium nitrate a month after planting. The field was rotated with maize and soybean in the past and had maize (2015) as last pre-crop before the experiment. The plants were at flowering stage when single plants were harvested on September 16, 4 months after sowing.

The setup and field management of the experiment in Changins (Switzerland) was described earlier [24]. The sampling was after 3 months while the plant was still at vegetative state, similarly to sampling in Reckenholz.

### Soil analysis

We determined the soil characteristics of the three fields at the Labor für Boden- und Umweltanalytik (Eric Schweizer AG, Thun, Switzerland). A fresh batch of Changins soil was re-analyzed for pH, soil texture and soil nutrients in parallel with the field soils from Aurora and Reckenholz. Soil chemical characteristics were determined in 1:10 water (H_2_O, proxy for plant available nutrients) and 1:10 acetate-ammonium EDTA (AAEDTA, proxy for reserve nutrients) extracts. Total iron was analyzed by weighing 250 mg of dried and ground soil samples in 50 ml centrifuge tubes, adding 4 ml of concentrated HNO_3_ (65%, subboiled) and following overnight incubation at room temperature 2 ml of H_2_O_2_ (35%, trace select™) was added. Samples were vortexed for 30 s and then heated for extraction for 30 min in a microwave oven at 95°C. For analysis the samples were diluted to 50 ml and centrifuged (5′ at 1200 rpm) and total iron (^56^Fe, ^57^Fe, analyzed in He mode) was analyzed by inductively coupled plasma mass spectrometry (ICP-MS, 7700x Agilent) using ^103^Rh and ^115^In as internal standards. Blanks were confirmed to contain <0.2% of the Fe concentrations in the samples. Table S2 documents the physico-chemical characteristics of the soils at the three locations Changins, Reckenholz and Aurora.

### Sample collection

The field experiment in Reckenholz was sampled using the same protocol as in Changins (see [24] for details). In brief, shoots were removed and a 20 × 20 × 20 cm soil core containing the root system was excavated, packed in plastic bags and transported to the laboratory for sample fractionation (Figure S1A). A destructive sampling was not possible in Aurora (plants were needed for another experiment), and therefore, we collected close to the base of the plants a cylinder (5 cm diameter × 20 cm depth, Figure S1B) containing soil and a part of the root system. Cylinders were also packed in plastic bags and transported to the laboratory for sample fractionation.

Samples were fractionated to the different compartments in the laboratory within maximum half a day after collection on the field. The collected soil cores or cylinders were broken apart to separate the roots from the soil. The soil fraction of the cores or cylinders was thoroughly mixed and a 2 ml aliquot, representing the soil compartment, was collected and frozen at −80°C until the further analysis. The root fraction was further processed on sterile Petri plates, and using sterile scissors, we collected a 10-cm root segment corresponding to a soil depth of −5 and −15 cm. On the Petri plates, we cut the root segment into small pieces and transferred them into sterile 50 ml Falcon tubes containing 25 ml of sterile Milli-Q water and we washed the rhizosphere from the roots by vigorously shaking the tubes 10 times. The roots were then transferred with sterile tweezers into fresh tubes containing 25 ml of sterile Milli-Q water for an additional wash step. This was repeated 4 times and we then transferred the washed roots into 15 ml Falcon tubes to freeze-dry them for 72 h. Lyophilized roots were then ground to a fine powder in a ball mill (Retsch GmBH, model MM301; settings 30 s at 30 Hz using on 1-cm steel ball) and aliquots, representing the root compartment, were transferred in 1.5 ml microcentrifuge tubes and stored at −80°C until further analysis. Of note, the sampling method for the root compartment does not discriminate between the inner root tissue and the root surface, and therefore, we refer to the sampling unit as “root microbiota.” The rhizosphere compartment was prepared by combining all four wash fractions (4× 25 ml) using centrifugation (5 min at 3220×*g*, discarding the supernatant), and the resulting pellets were collected in 1.5 ml microcentrifuge tubes and stored at −80 °C until further use.

### DNA extraction, PCR, and sequencing

DNA was extracted using the FastDNA SPIN kit for soil (MP Biomedical, USA) following the manufacturer’s instructions with 50–100 mg of ground roots, 100 mg rhizosphere soil, or 100 mg soil as input material. DNA concentrations were determined on a Varian Eclipse fluorescence plate reader (Agilent, USA) using the Quant-iT^TM^ PicoGreen® dsDNA Assay Kit (Invitrogen, USA) and a standard solution prepared from Herring Sperm DNA (Invitrogen, USA).

Bacterial and fungal community profiles were determined following the methodology described earlier [24]. In brief, bacterial profiles are based on PCR primers 799F [38] and 1193R [39] that span the hypervariable regions V5 to V7 of the 16S rRNA gene and fungal profiles are derived from the internal transcribed spacer (ITS) region 1 amplified with PCR primers ITS1F [40] and ITS2 [41]. The Additional file 2 contains the experimental design with the sample-to-barcode assignments. PCR reactions consisted of 5-prime HotMastermix (QuantaBio, USA) (1x), bovine serum albumin (BSA, 0.3%), forward primer (200 nM), reverse primer (200 nM), with 3 ng input DNA per reaction for soil and rhizosphere samples and 9 ng for root samples, completed to 20 μl with H_2_O. Each sample was amplified in 3 technical replicates and one control sample without DNA per barcoded primer combination. Cycling settings were 3 min at 94 °C for denaturation, 30 cycles (Bacteria: 30 s at 94°C, 30 s at 55°C and 30 s at 65°C; Fungi: 45 s at 94°C, 60 s at 50°C and 90 s at 72°C) and a third step of 10 min at 65°/72°C. Reaction triplicates were pooled and confirmed for absence of contamination by running an aliquot on a 1.5% agarose gel. PCR products were then purified with a PCR clean-up kit (Macherey-Nagel, DE), quantified with the PicoGreen assay as described above, equimolarly pooled, purified and concentrated with AMpure XP beads (Beckman Coulter Inc, USA) and quantified with Qubit (Thermo Fisher, USA).

Library preparation was completed by ligation of the Illumina adapters by the Functional Genomics Center Zurich (http://www.fgcz.ch/), where they were sequenced on a MiSeq instrument in paired-end 2 × 300 bp mode (Illumina, USA). The raw sequencing data were deposited at the European Nucleotide Archive (http://www.ebi.ac.uk/ena), see Table S1 for details linking libraries, MiSeq runs, ENA study accessions and sample IDs.

### Bioinformatics

The paired-end raw reads were processed according to the bioinformatic script and parameters therein as provided in the Additional file 3. Briefly, we trimmed the low quality ends of the sequence reads R1 and R2 by cutting the reads to 280 nt and at the same time we removed all reads shorter 100 nt or reads with >1 ambiguous nucleotides using PRINSEQ (0.20.4; Schmieder & Edwards, 2011). We merged the reads with a minimum overlap of 15 nt and a maximum overlap of 250 nt with FLASH (1.2.11; [42]). Individually barcoded samples were demultiplexed using Cutadapt (2.4; [43]) and filtered for GC content (range 30-70%) and quality (min. mean qual score = 20 and no ambiguous base calls) with PRINSEQ. We then used UNOISE3 (v11; Edgar, 2016a) to compute zero-radius operational taxonomic units (zOTUs), and we inferred taxonomy assignments with the Sintax algorithm [44] and the SILVA (v128, [45]) and UNITE (v7.2, [46]) databases for bacteria and fungi, respectively. We reported taxonomy assignments with a >0.85 confidence cutoff. Of note, the sequence data of the field experiment in Changins [24] were reprocessed and analyzed as zOTUs in this study. Table 1 summarizes the number of replicate microbiota profiles per compartment, field location, maize genetic background and genotype.

**Table 1:** Experimental design. Number of replicates in each sample group

Location	*Changins		Aurora				Reckenholz			
Background	B73		W22				B73		W22	
Genotype	WT	*bx1*	WT	*bx1*	*bx2*	*bx6*	WT	*bx1*	WT	*bx1*
Soil	10**		8	8	8^f^	8	11	12	6^f^	12
Rhizosphere	10	7	8	8	8	8	11	12	6^f^	12
Roots	10^b^	7^b^	6	7	6	8	11	12	6^f^	12

*Samples from Hu et al. (2018)

**Bulk soil sampling did not discriminate plant genotype

^b/f^Single bacterial or fungal profiles were removed from the analysis, because of low sequence numbers

### Microbiota analysis in R

All steps of the microbiota analysis were performed in R (version 3.5.1) and are documented together with all input files, parameter settings and functions required for replication of the analysis (https://github.com/PMI-Basel/Cadot_et_al_BX_microbiota). The analysis logic with the key steps is illustrated in Figure S2.

The Additional file 2 contains the metadata for each sample and is loaded into R as the experimental design. Bacterial and fungal zOTUs were renamed to bOTUs and fOTUs, respectively. Microbiota profiles were filtered to exclude zOTUs classified as eukaryotes, cyanobacteria or when assigned to plant mitochondria or chloroplasts. When inspecting the mean sequencing depths of the samples, we found significant differences among our groups of samples (Figure S3, Kruskal-Wallis test, *P* < 0.05). This was seen between different locations and different compartments and was especially prominent in the fungal data with up to a 6.3 fold difference in mean sequencing depth. We normalized bacterial and fungal count tables by subsampling that data to 9000 and 4000 sequences per sample, respectively, to avoid confounding our comparisons between locations and between compartments with differences in sequencing depth. Compared to other normalization techniques, rarefication mitigates artifacts of sampling depth more effectively, especially for datasets with low or uneven sequencing depth between groups [47]. For groups with large differences in the mean sequencing depth between groups, rarefying improves the clustering of samples according to biological origin, as well as decreasing the false discovery rate in differential abundance testing.

The rarefied count tables were utilized for alpha and beta diversity analyses using the R packages *phyloseq* [48] and *vegan* [49]. We utilized the R-package *edgeR* [50] for identification of differentially abundant zOTUs within location and plant compartment subgroups. We used the terms ‘enriched’ or ‘depleted’ for describing the direction of differential abundance and remind the reader that they must not be understood in absolute terms, but rather in the framework of relative abundance data (changes in relative abundances of one taxon are driven by changes of other taxa in the data). The likelihood ratio tests were performed on data that was filtered to contain ‘quantifiable’ zOTUs (we defined ‘quantifiable’ as zOTUs with minimal abundance according to the lowest replicate number per test group; e.g., 5 replicates, min. abundance 5 sequences), and we normalized the counts using the trimmed mean of *M* values method (TMM normalization, [51]). Of note, TMM normalization assumes a constant abundance of a majority of species. To avoid violating this assumption and because microbes are highly variable between locations or compartments (soil, rhizosphere and roots), we first split the data by location and compartment and then applied TMM normalization within these data subsets. *P* values were adjusted for multiple hypothesis testing [52]. For visualization, we expressed the TMM-normalized OTU abundances as percentages.

Fungal guild analysis was performed by comparing the taxonomies of the BX-sensitive fOTUs with the FUNGuild database [53]. This database uses the taxonomy of the fOTUs (done at genus or higher ranks) to assign ecological guilds (Saprotroph, Endophyte, Plant Pathogen, Animal Pathogen, Endomycorrhizal…) based on literature. We were mainly interested in the guild ‘Plant Pathogen’ and therefore simplified the divers’ ecological guilds by summarizing them as ‘others’. The functional assignments are ranked with confidence categories of ‘possible’, ‘probable’ and ‘highly probable’ of which we included all.

## Results

### Sequencing effort and general overview

In this study, we determined the microbiota profiles of the two field experiments in Aurora (USA) and Reckenholz (Switzerland) by sequencing amplicons of the 16S rRNA gene and the first internal transcribed spacer region for bacterial and fungal profiling, respectively. We analyzed this new data together with existing microbiota profiles from our earlier field experiment in Changins (Switzerland [24];). The whole analysis covers microbiota profiles of soil, rhizosphere and root compartments of WT and different mutant lines collected in Changins for the genetic background B73, in Reckenholz for B73 and W22, and in Aurora for W22 (Table 1). We performed the comparative microbiota analysis at the resolution of exact sequence variants, referred to as zero-radius operational taxonomic units (zOTUs), following the UNOISE method [54]. Bacterial community profiling yielded a total of 11,125,692 high-quality sequences (range 7541–161,133, median 41,111; Figure S3). Fungal community profiling yielded a total of 3,946,277 high-quality sequences, (range 4052–46,120, median 12,442; Figure S3). Rarefaction analysis showed that we sufficiently sampled the bacterial and fungal zOTUs (hereafter bOTUs and fOTUs, respectively) for most samples (Figure S4).

**Fig. 1:**
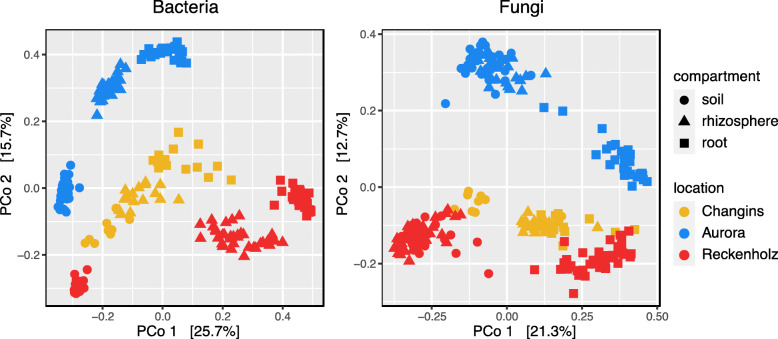
Microbiotas differ between compartments and locations. Unconstrained principle coordinate analysis (PCoA) of beta-diversity using Bray-Curtis distances of bacteria (left) and fungi (right) communities in root (circles), rhizosphere (triangles) and soil (squares) compartments from Changins (yellow), Aurora (blue) and Reckenholz (red) locations

To obtain an overview over the dataset, we first performed a general examination of the microbiota profiles from the different compartments and from the different locations (see Additional file 1: Supplementary Results, part 1). The taxonomy, alpha- and beta diversity analyses revealed that microbiotas differed strongly between the compartments as well as between locations (Figs. 1, S5-S6, Tables S3, S4, S5). This corroborates the work of previous studies that have shown a high context dependency of microbiotas from different compartments and/or locations [7, 16].

### Benzoxazinoid exudation shapes rhizosphere microbial communities

To answer the first research question—How do soils, rhizosphere and roots compare in their microbiota responses to BX exudation?—we analyzed the three compartments separately. We compared the taxonomy, alpha- and beta diversities limited to WT and *bx1* mutant lines, as these plant lines were present in all field experiments.

**Fig. 2:**
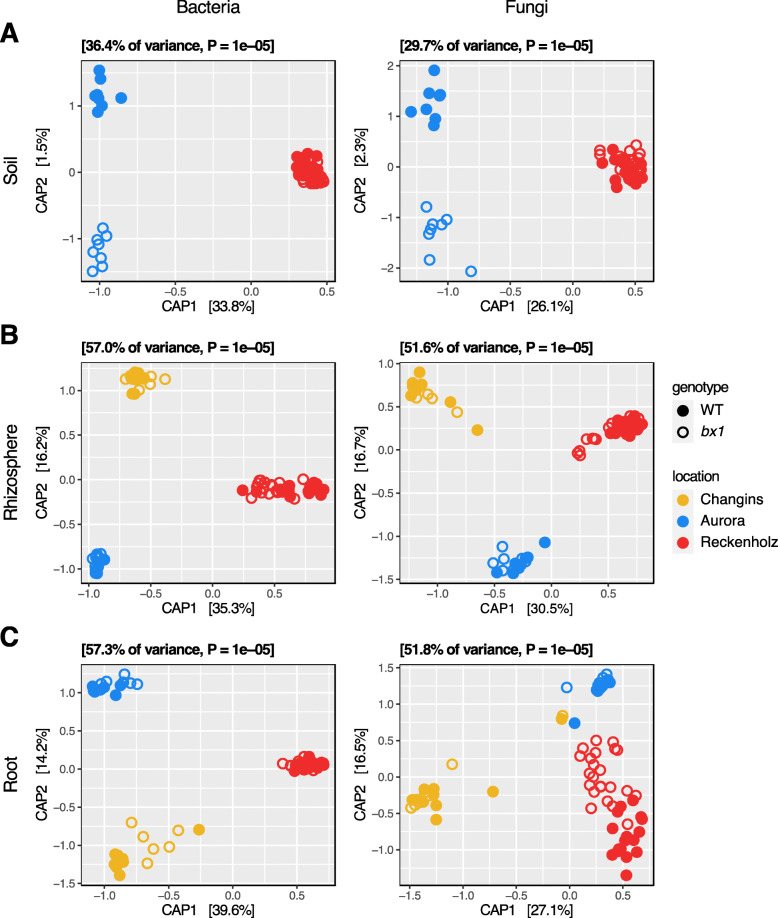
BX exudation impacts rhizosphere and root microbiotas. Compartment-wise Constrained Analysis of Principal Coordinates (CAP) using Bray-Curtis distances of community profiles from bacteria (left) and fungi (right). CAPs were performed using the model ‘~ *genotype* * *location’*. Wild-type (WT, filled) and *bx1* mutant (open) lines in **a** soil, **b** rhizosphere and **c** root compartments from Changins (yellow), Aurora (blue) and Reckenholz (red) locations

In our earlier work, we had compared the rhizosphere and root communities relative to the soil microbiota at whole field scale, but we did not specifically test whether BX exudation would also impact the soil microbiota in the soil cores that were used for the feedback experiments [24]. Therefore, we determined whether BX exudation would also impact the soil microbiota in the sampled soil cores of 20 × 20 × 20 cm from around WT and *bx1* plants. Comparing the phylum and family profiles, we found that the bacterial and fungal communities of the soil cores were similar between WT or *bx1* plants (Tables S6 & S7). The alpha and beta diversity analyses revealed that both soil bacterial and fungal Shannon diversity were unaffected by BX exudation (Fig. 2a, Tables S8 & S9).

In the rhizosphere compartment, however, the taxonomy analysis revealed numerous bacterial and fungal phyla and families differing in abundance between plants that do or do not secrete BXs (Tables S6 & S7). Although with statistic support in only one location, noteworthy is a consistent enrichment of Bacteroidetes in the rhizospheres of BX-deficient plants (WT, 7%, *bx1* 11% mean relative abundance; mainly represented by Flavobacteriaceae). Otherwise, the enrichments of other taxonomic groups were specific to each of the 3 field experiments (for details, see Additional file 1: Supplementary Results, part 2). Similar to the soil compartment, both bacterial and fungal alpha diversity in the rhizosphere were unaffected by BX exudation (Table S8). With regard to community composition, PERMANOVA quantified small yet significant effect sizes due to BX exudation of 2.2% for bacteria and 2.1% for fungi (Fig. 2b, Table S9). Slightly larger and also significant effect sizes were found for the interaction terms of BX exudation and location, suggesting condition-specific BX effects on the microbiota.

In roots, the taxonomic analysis detected a few differences between WT and *bx1* plants (Tables S6 & S7). The Bacteroidetes tended to be more abundant in roots of BX mutants at all locations again, while the identified significant enrichments of taxonomic groups were specific to each of the three field experiments (for details, see Additional file 1: Supplementary Results, part 2). The root bacterial diversity was unaffected by BX exudation, we found an enrichment in fungal Shannon diversity in WT compared to *bx1* root samples at all three locations (Table S8). BX exudation significantly impacted the root microbiota, with a more pronounced effect on the fungi (6.1%) than on the bacteria (1.6%; Fig. 2c, Table S9). The BX effects are condition-specific for the fungi (significant interaction of BX exudation and location) as seen with the strong effect in Reckenholz compared to the other locations. Also for bacteria the BX effects tend to be condition-specific as evidenced by the larger effect size of the interaction term and with the stronger effect seen in Changins compared to the other locations.

In conclusion, the soil microbiota in the collected 20 × 20 × 20 cm soil cores is largely unaffected by maize BX exudation, while community composition of rhizosphere and root compartments changes significantly depending on the specific field conditions.

### Effects of mutations in the BX biosynthesis pathway outweigh effects of plant genetic background

To answer the second research question—What is the influence of the plant genetic background on BX-mediated microbiota effects?—we studied the WT and *bx1* mutant lines in both genetic backgrounds, B73 and W22, in the Reckenholz field experiment.

**Fig. 3:**
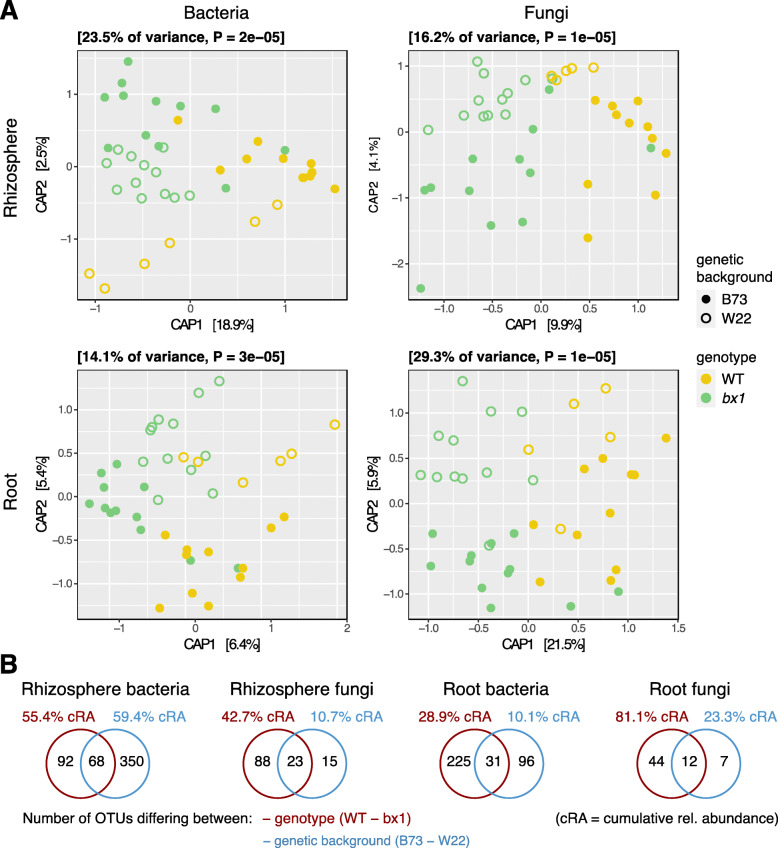
Microbiotas differ more by BX exudation than genetic background. Compartment-wise Constrained Analysis of Principal Coordinates (CAP) using Bray-Curtis distances of community profiles from bacteria (left) and fungi (right) and from rhizosphere (upper) and root compartment (down) from the Reckenholz experiment are shown in **a**. CAPs were performed using the model ‘~ *genotype* * *genetic background*. Genotypes of wild-type (yellow) and *bx1* mutant (green) lines differ in color while the genetic backgrounds B73 (filled) and W22 (open) distinguish by symbol filling. **b** Number of OTUs that differed significantly by the two factors *genotype* (WT vs. *bx1*) or *genetic background* (B73 vs. W22) as determined by edgeR analysis (FDR < 0.05, Table S10). The % cRA represents the cumulated relative abundance of all detected differentially abundant b/fOTUs for the respective statistic contrast term

Constrained Analysis of Principle Coordinates (CAP), using the model ‘~ *genotype* * *genetic background*’, visualizes the relative effect sizes of *genotype* and *genetic background* with *genotype* mainly separating along the CAP axes 1, and *genetic background* along the CAP axes 2. Supported by the exact quantifications, *genotype* (WT vs. *bx1*) explains more variation than *genetic background* (B73 vs. W22) for bacteria and fungi in both compartments (Fig. 3a). We confirmed the effect sizes of *background* and *genotype* relative to *compartment* employing PERMANOVA (Table S10). Compared to the major source of variation (*compartment* explains 35% in bacteria and 50% in fungi), maize *genetic background* (2.7%; 2.5%) explained less than *genotype* (3.4%; 4.5%) in both bacterial and fungal communities. Pairwise PERMANOVA indicated for both root and rhizosphere compartments that the microbiotas differed mostly between B73 and its mutant *bx1*, whereas this differentiation was weaker in the W22 background (Table S10). To further characterize the impacts of *background* and *genotype* on the rhizosphere and root microbial communities, we determined the number of b/fOTUs that differed significantly between these two factors. With the exception of the rhizosphere bacteria, we found fewer b/fOTUs differing between B73 and W22, while more b/fOTUs discriminated wild-type from mutant genotypes (Fig. 3b, Table S11). This result was also confirmed quantitatively with the differentially abundant b/fOTUs having higher cumulative relative abundances between genotypes compared to between backgrounds.

Taken together, the effects on microbial communities comparing the two genetic backgrounds are weaker than the effects of mutations in the BX biosynthesis pathway. The effect of the mutation (WT vs. *bx*1) appears more pronounced in B73 compared to W22.

### *BX2* and *BX6* show stronger similarity in their impact on microbiota structure compared to *BX1*

We then approached the third research question—How do different mutations in the BX biosynthesis pathway shape rhizosphere and root microbial communities?—by comparing the mutants *bx1*, *bx2* and *bx6* to the background W22, which were all grown under field conditions in Aurora. The *bx2* mutant has a BX accumulation profile similar to *bx1*, whereas *bx6* accumulates different BX species compared to the wild-type line W22 (see the “Introduction” section).

**Fig. 4:**
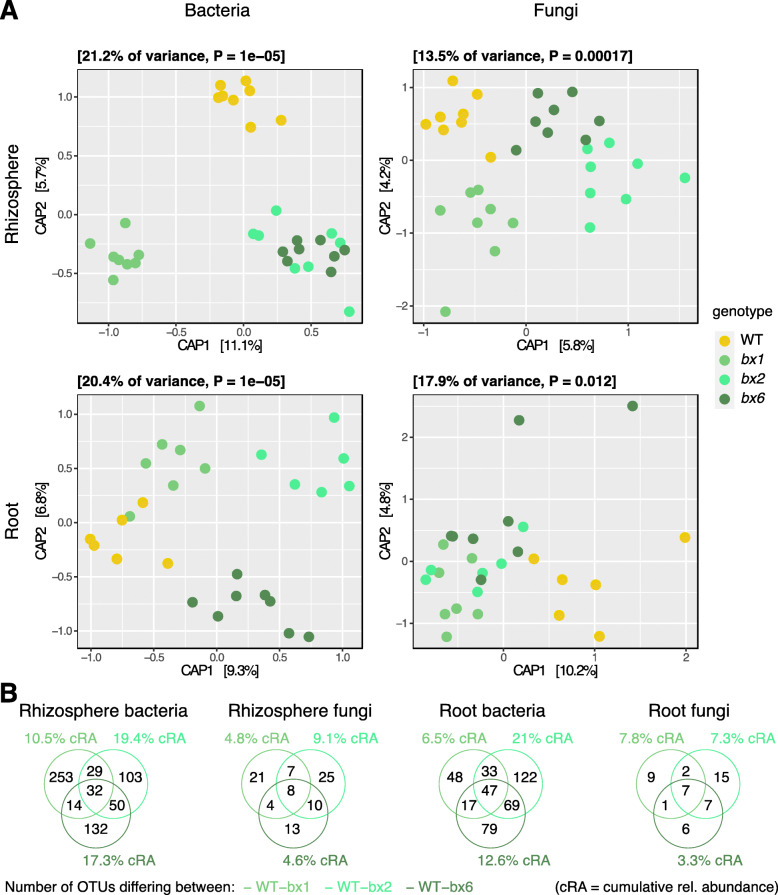
Microbiotas of *bx2* and *bx6* tend to be more similar to each other than *bx1*. Compartment-wise Constrained Analysis of Principal Coordinates (CAP) using Bray-Curtis distances of community profiles from bacteria (left) and fungi (right) and from rhizosphere (upper) and root compartment (down) from the Aurora experiment are shown in **a**. CAPs were performed using the model ‘~ *genotype*’. Genotypes differ in color with wild-type W22 in yellow, the *bx1* mutant in medium, the *bx2* mutant in light and the *bx6* mutant in dark green. **b** Number of OTUs that differed significantly between the wild-type line W22 and each mutant (same colors as in **a**) as determined by edgeR analysis (FDR < 0.05, Table S13). The % cRA represents the cumulated relative abundance of all detected differentially abundant b/fOTUs for the respective statistic contrast term

CAP analysis modeling *genotype* revealed that the mutant community profiles were all different from WT for both bacteria and fungi in both compartments (Fig. 4a). With the exception of the root fungi, the different mutants exhibited a mutant-specific clustering. An observation over CAPs of the rhizosphere was that the mutants *bx2* and *bx6* cluster closer to each other than to *bx1* or WT. PERMANOVA quantified overall variation among the genotypes of 8.2% and 6.4% for bacterial and fungal communities, respectively (Table S12). Pairwise PERMANOVA revealed significant differences between wild-type and mutant plants in bacterial communities in roots but not the rhizosphere. Fungal communities only differed significantly between wild-type and *bx1* roots as well as between wild-type and *bx2* rhizospheres.

To characterize the impacts of each mutation in the BX biosynthesis pathway on the rhizosphere and root microbial communities, we determined the b/fOTUs that differed significantly between wild type and each mutant (Table S13). We used the proportion of differentially abundant b/fOTUs (% among all OTUs of the analysis) to approximate the impact of each BX biosynthesis gene on microbiota structure (Fig. 4b). Lowest effect sizes for all three mutations were seen on the rhizosphere fungi. With the exception of the rhizosphere bacteria, a lack of BX2 appears to impact more microbes than a lack of BX1. And BX2 generally impacts more microbes compared to BX6. We found that the majority of b/fOTUs differed between individual mutants and the wild type, while only a small fraction of b/fOTUs discriminated all three mutants from W22 (Fig. 4b, Table S13). Inspecting the overlaps between mutants revealed that *bx1* and *bx6* shared the lowest fraction of bacteria (rhizosphere: 14 bOTUs; root: 17), *bx1* and *bx2* an intermediate fraction (29; 33) while most of the discriminating bOTUs were found shared by *bx2* and *bx6* (50; 69). This pattern was similar with the fungi and indicates that the biosynthesis genes *BX1* and *BX6* have more distinct impacts on microbiota structure, whereas *BX2* and *BX6* have more similarities in their effects on microbiota composition than *BX1* and *BX2*.

In conclusion, the three mutants discriminated from WT plants with mostly mutant-specific sets of differentially abundant b/fOTUs. Among these b/fOTUs, we noticed the subtle pattern that *bx1* and *bx2* sharing few while *bx2* and *bx6* sharing a large part of the discriminating b/fOTUs suggesting that the BX biosynthesis genes *BX2* and *BX6* have a more similar impact on microbiota structure than *BX1*.

### BX-sensitive microbes are condition specific

To answer the fourth research question—Is there a core group of microbial taxa that consistently responds to BX exudation across the different experiments?—we compared the wild types and mutants grown in the 3 field experiments in Changins, Aurora and Reckenholz. As a note of caution, this comparison is not limited to soil properties but encompasses all various conditions differing in these three experiments (e.g., climate, pre-planting, sampling time, fertilization and sequencing runs). We restricted these comparisons to WT and *bx1* mutants, as these plant lines were present at all locations. We excluded the W22 lines at the Reckenholz location because of unbalanced sample numbers. We searched for BX-sensitive b/fOTUs—being differentially abundant zOTUs between BX producing and defective plants—using likelihood ratio tests as implemented in edgeR [50].

**Fig. 5:**
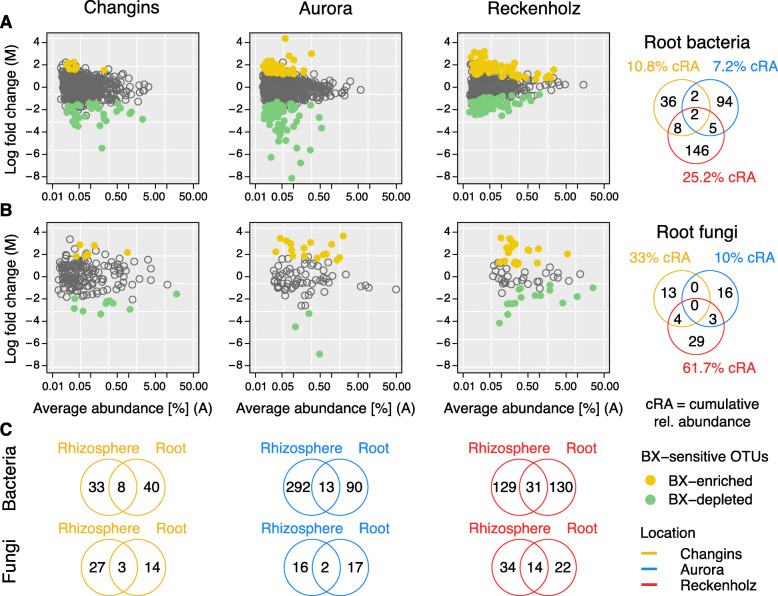
BX-sensitive root microbes are location-specific. The MA plots display the log-fold change (M) of all b/fOTUs and the average abundance (A, in log count per million) plotted on *y*- and *x*-axes, respectively. b/fOTUs being differentially abundant between wild-type and *bx1* mutant lines (BX-sensitive OTUs) were determined by edgeR analysis (FDR < 0.05, Table S14). Colors refer to enriched b/fOTUs in wild-type (yellow) or *bx1* mutant (green) lines; **a** reports the root bacteria and **b** the root fungi at the locations Changins (yellow), Aurora (blue) and Reckenholz (red). The comparison of BX-sensitive b/fOTUs between locations is visualized with the Venn diagrams. The % cRA represents the cumulated relative abundance of all detected differentially abundant b/fOTUs at each of the locations; **c** visualizes the comparison of BX-sensitive microbes detected in roots with the ones detected in the rhizosphere for each location (displayed in Figure S7)

In roots, we found 48 BX-sensitive bOTUs (with 10.75% cumulative relative abundance) and 17 BX-sensitive fOTUs at the Changins location (32.95%), 103 bOTUs (7.18%) and 19 (10%) fOTUs in Aurora and 161 bOTUs (25.21%) and 36 fOTUs (61.71%) in Reckenholz (Fig. 5a, b, Table S14). The majority of BX-sensitive bOTUs and fOTUs were specific to the each of the three experiments, with only a few being shared between experiments. The same finding applies also to the rhizosphere compartment, where a similar condition-specific pattern of BX-sensitive bOTUs and fOTUs was seen (Figure S7, Table S14). Comparing the BX-sensitive OTUs between rhizosphere and root samples in each experiment revealed that the majority were specific to each compartment while the minority was shared between them (Fig. 5c).

**Fig. 6:**
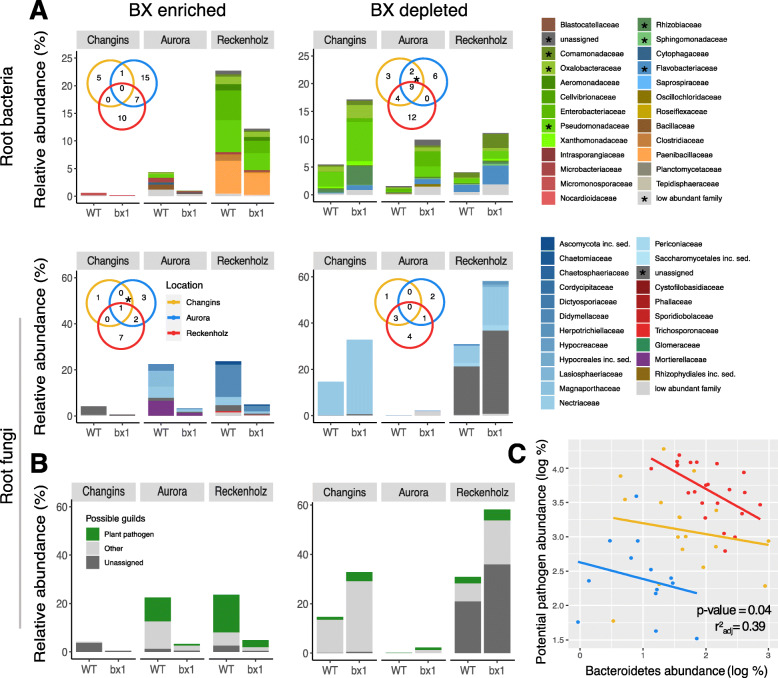
Taxonomic pattern of BX-sensitive root microbes. The barplots depict the mean relative abundances (in %) for each location and taxonomies of all root bOTUs (upper panels) and root fOTUs (lower panels) that differed significantly in abundance between wild-type (WT) and *bx1* mutant lines (i.e. the BX-sensitive b/fOTUs as determined by edgeR analysis, FDR < 0.05, Table S14). The BX-enriched (left panels) and BX-depleted taxa (right panels) correspond to the same yellow (enriched in WT) and green (enriched in *bx1*) b/fOTUs of Fig. 5a, respectively. Individual b/fOTUs are displayed in a stacked manner sorted by their taxonomic assignment at family level. The Venn diagram insets compare the family assignments of the BX-sensitive taxa between the locations Changins (yellow), Aurora (blue) and Reckenholz (red). Overlapping family assignments are indicated with asterisks in the plot and marked in the taxonomy legend; **b** visualizes the proportion of assignments to ‘plant pathogen’ among the FUNGuild annotations. The sets of BX-enriched and BX-depleted root fOTUs from each location were annotated individually to their ecological guilds. **c** Abundance of bOTUs belonging to the class Bacteroidetes displayed versus the abundance of potential pathogenic fungi in the roots. ANCOVA on these variables with locations as covariate was performed, and *p* value and *r*^2^ are displayed

We then inspected the BX-sensitive bOTUs and fOTUs, which were specific in each experiment, for common taxonomic patterns when being enriched or depleted by BX exudation (Figs. 6a, S8, Table S14). We did not find evidence that any bacteria or fungi of certain taxonomic families that were consistently enriched by BX-producing plants in the roots or in the rhizospheres in all 3 experiments. Nevertheless, we inspected, the abundance of *Methylophilaceae* in the Reckenholz and Aurora experiments, because such OTUs were previously found to be enriched by BXs in experiments conducted with Changins [24] and Sheffield soils [25]. We confirmed the BX-enrichment of a *Methylophilales* (bOTU479) and 2 *Methylophilaceae* (bOTU270 and 647) in the rhizosphere in Changins (Figure S9). It is noteworthy that bOTU270 and bOTU479 were also significantly enriched in the rhizosphere samples in Reckenholz, whereas bOTU647 was significantly enriched by BXs in roots in the Aurora experiment (Table S14).

While a taxonomic pattern was not found among BX-depleted fungi, we found that the bOTUs of same taxonomic families were consistently depleted from roots of BX producing plants in all 3 experiments (Fig. 6a). BX-depleted root bacteria were commonly assigned to families including the *Pseudomonadaceae*, *Oxalobacteraceae*, *Flavobacteriaceae*, *Rhizobiaceae*, *Comamonadaceae* and *Sphingomonadaceae*. In agreement with this observation, *Flavobacteriaceae* and *Comamonadaceae* were also consistently depleted in rhizosphere samples of WT plants in all 3 experiments (Figure S8A). Other than this common taxonomic pattern of BX-depleted bacteria, most of the BX-dependent enrichments and depletions were condition-specific (Table S14, see Additional file 1: Supplementary Results, part 3).

Because of earlier reports [25, 26], we investigated whether microbiotas of BX producing plants would comprise fewer fungal species with taxonomic links to plant pathogens. We used FUNGuild to annotate the fOTU data to ecological guilds [53] and identified fOTUs with possible assignments to plant pathogens among the fOTUs that were BX-enriched or BX-depleted. Over all three experiments, while we found only few and non-abundant BX-sensitive fOTUs in the rhizosphere samples (Figure S8B), we observed more fOTUs with possible links to plant pathogens in roots. These fOTUs were particularly abundant on BX-producing plants in Reckenholz and Aurora (Fig. 6b). This observation suggested that wild-type maize plants attract fungal pathogens by BX exudation. In addition, we explored the relationship in the roots between potential pathogenic fungi and the bacterial class Bacteroidetes, which includes the Flavobacteria and Chitinophaga families that were shown to be enriched upon fungal infection [55] and may have a protective effect (Fig. 6c). By performing an ANCOVA regression, we found out the negative relationship to be significant (*p* < 0.05, *r*^2^ = 0.39).

Taken together, the exudation of BXs affects different sets of bacteria and fungi in the different field experiments and in the different compartments. While BX-producing plants did not consistently enrich microbes of certain taxonomic lineages in their roots or rhizospheres, we found that BX exudation depleted bacteria assigned to *Flavobacteriaceae* and *Comamonadaceae* from root and rhizosphere compartments, as well appearing to enrich for plant-pathogenic fungi.

## Discussion

In our earlier work, we showed that the secretion of BXs by a first generation of maize plants can drive microbial feedbacks on growth and defense in the next generation of maize plants [24]. The mechanistic basis for these feedbacks was that the secretion of BXs altered the structure of root and rhizosphere microbial communities. Such BX impacts on microbiotas were confirmed in young plants [25] and in semi-artificial rhizobox systems [26]. However, it was unclear whether such effects also are consistent under real world conditions. To study conserved microbiota response patterns to BX exudation, we profiled soil, rhizosphere and root microbial communities in two additional field experiments in Aurora and Reckenholz, which we conducted in a manner similar to the first one in Changins [24].

### Genetic background

Microbiota profiles of the WT and *bx1* mutant plants differed in the genetic backgrounds B73 [24] and W22 [25, 26]. We grew B73 and *bx1*(B73) in parallel with W22 and *bx1*(W22) to learn: How do genetic background and the *bx1* mutation compare in their effects on microbial communities? While B73 and W22 differ at a multitude of genetic loci, the genetic variation between wild-type and mutant lines is low, as they differ mainly in the *BX1* gene, which defines their in-/ability of BX synthesis. The *bx1* mutation in B73 represents a near-isogenic line based on five backcrosses in this genetic background [35], while *bx1*(W22) contains a single *Ds* transposon insertion compared to W22 [37]. Ordination analysis (Fig. 3a), variation partitioning (Table S10) and the number of differentially abundant OTUs between these two factors (Fig. 3b, Table S10) revealed that the presence/absence of a functional *BX1* gene had more impact on roots and rhizosphere microbiotas than other differences between the B73 and W22 backgrounds. Multiple genetic differences, such as between lines of different backgrounds, plant varieties or accessions, typically account for ~5–6% of variation in root and rhizosphere microbiota composition [7, 16]. Since, we found a similar level of microbiota variation (Table S10) comparing plants that are genetically nearly identical but differ in a discrete chemical pathway, this suggests that the BX exudation presents a key trait by which maize plants structure their root and rhizosphere microbiota.

The effect of the mutation (WT vs. *bx1*) appeared more pronounced in B73 compared to the background W22 (Table S10). The multiple genetic differences between the two backgrounds serve as the obvious explanation for this observation whereas, mechanistically, this could be due to quantitative differences in the amounts of BXs that are exuded by these two backgrounds. The W22 background produces about 15% lower levels of total BXs in the roots compared to B73 [31] and therefore possibly structures its root and rhizosphere microbiota to a lesser extent than B73 does. Specific work is needed to validate quantitative BX effects on microbiota composition, for instance by studying BX overexpression lines [56].

### Microbiotas of BX biosynthesis mutants

Studying the broader impact of BXs exudation on the root and rhizosphere microbiota, we hypothesized that variations in microbiota composition will be consistent with the BX accumulation patterns of the mutants. The *bx1* and *bx2* mutants secrete very low levels of BXs whereas *bx6* secretes different BXs (see the “Introduction” section for details). Consistent with our hypothesis, the microbiota profiles of BX-producing WT plants would differ most strongly compared to the mutants *bx1* and *bx2*, and the profiles of the two mutants would share similarities with each other. We then saw two scenarios for *bx6* in this hypothesis: (a) if the total amount of secreted BXs matters, the microbiota profiles of *bx6* would be like WT plants or (b) if the speciation of BXs is relevant, *bx6* would display an intermediate or different microbiota composition compared to WT, *bx1* and *bx2* plants.

We found all mutant microbiota profiles to be different from WT microbiota profiles (Fig. 4a, Table S12). In roots, the fungi did not differ between the mutants, whereas mutant-specific profiles were found for root bacteria. In the rhizosphere, the fungi revealed mutant-specific profiles and the bacteria were similar between *bx2* and *bx6* but different from WT and *bx1*. In all CAPs, a closer clustering of the mutants *bx2* and *bx6* compared to *bx1* or WT was observed. The analysis of the number of differentially abundant b/fOTUs (each mutant vs. WT) and their overlap between mutants revealed that the biosynthesis genes *BX1* and *BX6* have the least and *BX2* and *BX6* share most similarities in their effects on microbiota composition (Fig. 4b, Table S13). Hence, we rejected the first part of the aforementioned hypothesis (stating that overlap is expected between *BX1* and *BX2*) and concluded that factors additional to the BX accumulation pattern explain the microbiota profiles between the mutants. Regarding the second part of the hypothesis, we concluded that because the microbiota of *bx6* was found to be different compared the wild-type plants and that the speciation of BXs is relevant for root and rhizosphere microbiota assembly.

Coming back to additional factors than BX accumulation which could explain the microbiota profiles between the mutants: benzoxazinoids and their precursors function as signaling molecules in cereals [57–59] and may regulate the production of other secondary metabolites. Wheat plants that overexpress a maize methyl-transferase which shifts benzoxazinoid production from DIMBOA-Glc to HDMBOA-Glc also accumulate higher amounts of ferulic acid in the leaves [60]. Furthermore, Cotton et al. (2019) found differences in metabolic features in the roots of *bx1*, *bx2* and *bx6* mutants that could not be linked to BXs, but matched *m*/*z* values of flavonoids. Correlation analysis between microbes and metabolites revealed that, although the abundance of BX-stimulated OTUs correlated positively with BXs, the same OTUs also correlated positively with other features. Half of the features which correlated positively with BX-stimulated OTUs were assigned to potential flavonoids. Given that flavonoids can function as a semiochemical for root bacteria [61], it was suggested that these metabolites, possibly jointly with BXs, structure the root microbiota. More work such as untargeted metabolomics comparing the exudates of BX mutants and wild types in both backgrounds is required to assess the contribution of BX-dependent metabolites as well as other metabolites, which may be masked by BXs, for their role in structuring the root microbiota.

With respect to the mutants *bx1*, *bx2* and *bx6* in the W22 background, there is the particularity that the genes at the transposon launch sites participate in the flavonoid pathway. Both launch sites affect the downstream anthocyanin synthesis, but while R1 (*bx1* and *bx6* lines) is a bHLH transcription factor that could be acting pleiotropically, A1 (launch site for *bx2* line) is a structural gene downstream in the pathway [62]. However, the genetics of these launch sites is unlikely to drive BX-dependent microbiota effects, as a differential assembly was also found in the B73 background, which is unaffected in the flavonoid pathway. Our results are congruent with the hypothesis that BXs may act directly as well as indirectly via precursors or other metabolites to shape rhizosphere microbiota.

### BX-sensitive microbes in different experimental conditions

The three field experiments differed in multiple environmental conditions (soil properties, climate, etc.) as well as in their experimental setups (pre-planting, sampling time, fertilization or sequencing runs). Despite these differences, we were interested in testing whether there is a core group of microbial taxa that consistently responds to BX exudation across the different conditions in the three field experiments. We were less interested in microbiota differences because, for instance soil properties are a well-known primary determinant of microbial community structure [7, 16]. Consistently, we found that most BX-sensitive microbes, whether enriched or depleted, were specific for one of the three experiments (Fig. 5, Table S14). This was already seen earlier when comparing the BX-sensitive taxa in Changins soil [24] to the ones detected in Sheffield soil [25]. Considering the condition-specificity of BX-effects on the root and rhizosphere microbiota, it appears plausible that the exudation quantities of BXs also may vary depending on the conditions, i.e. fertilization or the local biogeochemical environment. We hypothesize that environmentally regulated exudation of BXs could be linked to the phytosiderophore function of BXs, as these root-secreted compounds complex iron for plant uptake [31]. We need further work to clarify whether maize plants adjust the levels of BX exudation in a context-dependent manner and eventually in response to the levels of available iron in soil. Note that in order to work out the impact of specific biogeochemical properties of a soil and their impact on BX exudation, this would require controlled conditions (climate, soil moisture, homogenous fertilization levels, etc.) and harvest of plants at multiple timepoints across the season.

The finding that BX-sensitive microbes are condition-specific, with only a few being shared between experiments (Fig. 5, Table S14), rules out that BXs would selectively enrich or deplete the same microbial species across different soils and experiments. Although they are not enriched consistently at each location and in each compartment, we found a significant enrichment of *Methylophilaceae* in rhizosphere samples in Reckenholz and in roots in Aurora from BX-producing plants (Figure S9, Table S14), similar to the experiments conducted with soils in Changins [24] and Sheffield [25]. The detection of the same sequence variants (at the level of individual zOTUs) in experiments with different soils on European and American continents makes it unlikely that it derived from soil and points to the possibility that these bacteria originate from the maize seed material. Some methylotrophic bacteria such as *Methylobacteria* (*Methylobacteriaceae*, being Alphaproteobacteria) or *Methylophilus* (*Methylophilaceae*, being Betaproteobacteria) have been reported as maize seed endophytes [63–66]. Future experiments, e.g. by sequencing kernels or roots from plants grown in sterile substrate or culturing seed endophytes on media with methanol as sole carbon source, are needed to clarify the endophytic presence of *Methylophilaceae* bacteria in seeds of BX-producing maize lines.

When inspecting the BX-sensitive microbes at higher taxonomic rank, we noticed that BX-producing plants consistently depleted bacteria assigned to the *Flavobacteriaceae* and *Comamonadaceae* from their roots or rhizospheres (Fig. 6). A similar pattern was seen among the few BX-sensitive OTUs in 17-day-young plants, with the majority being depleted while a smaller fraction was enriched by BXs [25]. Of note, only the fraction that was depleted included *Flavobacteriaceae* and *Comamonadaceae*. Besides depletion of *Flavobacteriaceae*, they also found that other Bacteroidetes bacteria such as *Cytophagaceae* and *Chitinophagaceae* were negatively affected by BX exudation. We also noticed in our dataset that *Cytophagaceae* were depleted by BX exudation in Changins and Aurora locations (Table S14). Together with the observation that bacterial OTUs assigned to the phylum Bacteroidetes tended to be depleted by BX exudation also in an earlier study [26], this suggests an overall negative impact of BX exudation on Bacteroidetes bacteria.

While we did not observe that BX-producing plants would consistently enrich certain taxonomic groups of microbes to their roots or rhizospheres, we found evidence for an enrichment of potential fungal pathogens in the FUNGuild analysis (Fig. 6b). FUNGuild indicated more fOTUs in roots with possible links to plant pathogens, and they were particularly abundant on BX-producing plants in Reckenholz and Aurora. A FUNGuild analysis has the limitations that the ecological guild is inferred from taxonomy data and that such a functional assignment fails to account for intra-species functional variation [53]. Therefore and because our observation is in contrast with earlier findings [25, 26], our observation that wild-type maize plants may attract fungal pathogens by BX exudation and thus requires further and methodologically independent work (e.g. using shotgun metagenomics).

The enrichment of potential fungal pathogens and the depletion of *Flavobacteria* and other Bacteroidetes bacteria by BX exudation is interesting considering that sugar beet plants specifically enrich bacteria of two genera of the Bacteroidetes in response to fungal infection [67]. Carrion et al. (2019) reported that *Chitinophaga* and *Flavobacteria* have characteristic chitinase genes and previously unknown biosynthetic gene clusters encoding secondary metabolites that are essential for disease suppression. Consistent with their discovery, we also observed an antagonistic relationship between Bacteroidetes bacteria and fungal pathogens in all three locations, in the roots: Bacteroidetes abundance negatively correlated with potential fungal pathogen abundance (Fig. 6c). Future work is needed to disentangle whether direct microbe-microbe interactions govern this antagonistic relationship or whether the BX function as driving force with possible repellent and attractive activities on Bacteroidetes bacteria and pathogenic fungi, respectively.

### BX effects on microbiota in soil cores

In addition to the research questions on genetic background, mutants and soil properties, we wanted to close the knowledge gap whether the microbiota in the soil cores might also be affected by BX exudation. This question remained open from our earlier work, where we had compared the rhizosphere and root communities relative to the soil microbiota at field scale, which was analyzed based on random soil samples collected from across the field [24]. However, the microbial feedback experiments were conducted using 20 × 20 × 20 cm soil cores that we had not specifically tested for eventual effects of BX exudation. Therefore, we analyzed DNA samples from such soil cores taken in the fields of Reckenholz and Aurora and found that the soil microbiota in the soil cores was largely indistinguishable, irrespective of whether the soil cores were collected from WT or *bx1* plants (Fig. 2a, Tables S6, S7, S8, S9). This finding suggests that the amount of BXs, which was secreted into a 20 × 20 × 20 cm soil volume, becomes diluted to such an extent that BX-dependent microbiota differentiation is no longer seen. Nevertheless, we know that the population of microbes in the soil cores provokes a robust effect on the next generation of maize [24]. Possible interpretations are that, although no BX-dependent compositional differentiation is seen at the scale of whole soil cores, that the microbiota may be affected at a finer level, such as for example by adaptation or that such cores may contain aggregates of former BX rhizospheres, as well as also root fragments from the previous plant generation, and that these BX microbiota hotspots suffice to trigger the observed feedback.

## Conclusions

Our findings that exuded BXs may function in controlling the abundance of Bacteroidetes bacteria and fungal pathogens together with the positive feedback effects on plant health [24] and positions the BX pathway as an interesting target to maintain or further enhance in breeding programs. Incorporating plant-microbiome interactions into breeding programs relies on plant loci that explain heritable traits of plant microbiomes. The big challenge remains to identify plant genes that, beyond the strong environmental influences by soil properties, climate or field management, are responsible for the abundance of certain beneficial taxa or the expression of beneficial microbiome traits. This is a demanding task, as the heritability of plant microbiota composition is notoriously low [7, 16]. Even the most comprehensive analysis of plant microbiota heritability, although identifying a few taxa with genetically explained abundance, concluded that their heritabilities were low [55, 68]. This analysis included close to 5000 maize rhizosphere microbiota profiles from the 27 genetically diverse maize inbred lines that were planted in partly repeated field experiments at three sites to include variation in environment and time. Comparisons between genetic lines, plant varieties or accessions, which differ from each other genetically by a multitude of allelic variants, typically account for ~5–6% of variation in microbiota composition [7, 16]. We found a similar level of microbiota variation comparing plants that are genetically nearly identical but differ in a discrete major chemical pathway (Table S10). We argue that, compared to unbiased genetic diversity screens, studying candidate root exudate pathways will be a more promising approach to find host loci with heritable effects on the plant microbiota.

## Supplementary Information

**Additional file 1: Supplementary Results. Figure S1.** Setup of field experiments. **Figure S2.** Analysis steps. **Figure S3.** Sequencing effort by sample groups. **Figure S4.** Sampling intensity analysis. **Figure S5.** Taxonomy. **Figure S6.** Alpha diversity. **Figure S7.** BX-sensitive rhizosphere microbes across locations. **Figure S8.** Taxonomic patterns of BX-sensitive rhizosphere microbes. **Figure S9.** Abundance of Methylophilaceae bOTUs across compartments and locations. **Table S1.** Deposition of raw sequence data. **Table S2.** Soil characteristics in each location. **Table S3.** Taxonomic analysis comparing locations and compartments. **Table S4.** Alpha diversity analysis comparing locations and compartments. **Table S5.** Beta diversity analysis comparing locations and compartments. **Table S6.** Taxonomic analysis (phylum level) comparing BX effects in each compartment. **Table S7.** Taxonomic analysis (family level) comparing BX effects in each compartment and location. **Table S8.** Alpha diversity analysis comparing BX effects in each compartment. **Table S9.** Beta diversity analysis comparing BX and location effects in each compartment. **Table S10.** Beta diversity analysis comparing genetic background and genotype effects in each compartment (Reckenholz experiment). **Table S11.** zOTUs differing by genetic background or genotype (Reckenholz experiment). **Table S12.** Beta diversity analysis comparing mutant genotypes in each compartment (Aurora experiment). **Table S13.** zOTUs differing by mutant genotypes (Aurora experiment). **Table S14.** BX-sensitive zOTUs across all locations.

**Additional file 2: **Experimental design with the sample-to-barcode assignments.

**Additional file 3: **Bioinformatic script.

## Data Availability

The raw sequence data generated of this study are available from the European Nucleotide Archive repository [http://www.ebi.ac.uk/ena, see Table S1 for ENA study accessions and sample IDs]. All analysis code to reproduce this study is included in this published article [Additional file 2 contains the bioinformatic script] and a github project [https://github.com/PMI-Basel/Cadot_et_al_BX_microbiota] documents the microbiota analysis in R.
