# Supplementary material for: Specific and conserved patterns of microbiota-structuring by maize benzoxazinoids in the field
Source: Microbiome. 2021 May 7;9:103. doi: 10.1186/s40168-021-01049-2 (PMC8106187; doi:10.1186/s40168-021-01049-2)
Supplement: Supplementary file 2 — Additional file 1: Supplementary Results. Figure S1. Setup of field experiments. Figure S2. Analysis steps. Figure S3. Sequencing effort by sample groups. Figure S4. Sampling intensity analysis. Figure S5. Taxonomy. Figure S6. Alpha diversity. Figure S7. BX-sensitive rhizosphere microbes across locations. Figure S8. Taxonomic patterns of BX-sensitive rhizosphere microbes. Figure S9. Abundance of Methylophilaceae bOTUs across compartments and locations. Table S1. Deposition of raw sequence data. Table S2. Soil characteristics in each location. Table S3. Taxonomic analysis comparing locations and compartments. Table S4. Alpha diversity analysis comparing locations and compartments. Table S5. Beta diversity analysis comparing locations and compartments. Table S6. Taxonomic analysis (phylum level) comparing BX effects in each compartment. Table S7. Taxonomic analysis (family level) comparing BX effects in each compartment and location. Table S8. Alpha diversity analysis comparing BX effects in each compartment. Table S9. Beta diversity analysis comparing BX and location effects in each compartment. Table S10. Beta diversity analysis comparing genetic background and genotype effects in each compartment (Reckenholz experiment). Table S11. zOTUs differing by genetic background or genotype (Reckenholz experiment). Table S12. Beta diversity analysis comparing mutant genotypes in each compartment (Aurora experiment). Table S13. zOTUs differing by mutant genotypes (Aurora experiment). Table S14. BX-sensitive zOTUs across all locations. [file 40168_2021_1049_MOESM2_ESM.zip › Cadot_et_al_supplementary_information_revised_3.1_ESM.pdf]

ADDITIONAL FILE 1

Cadot et al. (2021, Microbiome Journal)

Title: Specific and conserved patterns of microbiota-structuring by maize benzoxazinoids in the field

INDEX:

SUPPLEMENTARY RESULTS

Part 1 | Analysis of 'compartment' and 'location' effects
Part 2 | Compartment-wise taxonomy analysis of 'BX exudation effects'
Part 3 | Analysis of BX-sensitive microbes

SUPPLEMENTARY FIGURES

Figure S1 | Setup of field experiments
Figure S2 | Analysis steps
Figure S3 | Sequencing effort by sample groups
Figure S4 | Sampling intensity analysis
Figure S5 | Taxonomy
Figure S6 | Alpha diversity
Figure S7 | BX-sensitive rhizosphere microbes across locations
Figure S8 | Taxonomic patterns of BX-sensitive rhizosphere microbes
Figure S9 | Abundance of Methylophilaceae bOTUs across compartments and locations

SUPPLEMENTARY TABLES:

Table S1 | Deposition of raw sequence data
Table S2 | Soil characteristics of each locations
Table S3 | Taxonomic analysis comparing locations and compartments
Table S4 | Alpha diversity analysis comparing locations and compartments
Table S5 | Beta diversity analysis comparing locations and compartments
Table S6 | Taxonomic analysis (phylum level) comparing BX effects in each compartment and location
Table S7 | Taxonomic analysis (family level) comparing BX effects in each compartment and location
Table S8 | Alpha diversity analysis comparing BX effects in each compartment
Table S9 | Beta diversity analysis comparing BX and location effects in each compartment
Table S10 | Beta diversity analysis comparing genetic background and genotype effects in each compartment (Reckenholz experiment)

Table S11 | zOTUs differing by genetic background or genotype (Reckenholz experiment)
Table S12 | Beta diversity analysis comparing mutant genotypes in each compartment (Aurora experiment)
Table S13 | zOTUs differing by mutant genotypes (Aurora experiment)
Table S14 | BX-sensitive zOTUs across all locations

SUPPLEMENTARY RESULTS

Part 1 | Analysis of 'compartment' and 'location' effects

We examined the microbiota profiles for 'compartment' and 'location' effects by analyzing the dataset based on taxonomy, alpha- and beta diversity.

Community taxonomy differs by locations and compartments

In a first step, we examined the taxonomy profiles at phylum level (**Table S3**). On average over all locations, the bacterial root microbiota was significantly enriched in Gammaproteobacteria (soil 7%, rhizosphere 26%, roots 33%), Actinobacteria (soil 10%, rhizosphere 6%, root 17%), Alphaproteobacteria (soil 8%, rhizosphere 14%, roots 13%) and Firmicutes (soil 0.5%, rhizosphere 1%, root 5%, **Fig. S5**). In contrast, Chloroflexi (soil 10%, rhizosphere 3%, root 2%), Deltaproteobacteria (soil 7%, rhizosphere 3%, root 1%), Acidobacteria (soil 7%, rhizosphere 2%, root 0.3%), Verrucomicrobia (soil 6%, rhizosphere 2%, root 0.2%) and Gemmatimonadetes (soil 7%, rhizosphere 1%, root 0.3%) were significantly more abundant in soil (**Fig. S5**). Wheras the taxonomic composition differed little between soils from different locations, we noticed more variation in the rhizosphere and root compartments. The rhizosphere samples in Reckenholz had a high proportion of Gammaproteobacteria, while Changins and Aurora were characterized by pronounced Bacteroides and Betaproteobacteria, respectively. In roots, high proportions of Actinobacteria were found in Changins, whereas Gammaproteobacteria were abundant in Reckenholz and Alphaproteobacteria were typical for Aurora (**Fig. S5**).

For the fungal microbiota, over all locations Ascomycetes (soil: 52%, rhizosphere: 51%, root: 86%) and Basidiomycetes (soil: 6%, rhizosphere: 8%, root: 8%) were significantly more abundant in roots (**Fig. S5**, **Table S3**). Soil fungal communities were significantly enriched in Mortierellomycota (soil: 20%, rhizosphere: 21%, root: 2%) and Glomeromycota (soil: 5%, rhizosphere: 1%, root: 2%) and they differed only marginally between locations. Rhizosphere communities in Aurora and Reckenholz also contained abundant Mortierellomycota whereas Basidiomycota were the second most abundant fungi in Changins. Root fungal communities were dominated by Ascomycetes at all locations (Changins: 61%, Aurora: 64%, Reckenholz: 64%). Some Basidiomycota and Glomeromycota fungi

were also found enriched in roots from Changins, while they were not much detected in Reckenholz and Aurora (**Fig. S5**).

Taken together, the taxonomic composition of the microbiotas differs strongly between soil, rhizosphere and roots as well as between the locations Changins, Aurora and Reckenholz.

Community diversity varies by compartment and is location specific

To investigate alpha diversity of the microbial communities, we rarefied the bacterial and fungal datasets to even sampling depths of 9,000 and 4,000 sequences, respectively, and statistically compared the Shannon metrics between compartments and locations (**Table S4**). Consistent with the rarefaction analysis (**Fig. S4**), bacterial diversity decreased from soil to rhizosphere to roots in all locations, was similar in the soil compartment at all three locations, but was found to be systematically lower in the rhizosphere and root compartments in Reckenholz soil (**Fig. S6**). Similarly, fungal diversity was high in soil and rhizosphere compartments and decreased in roots at all three locations (**Fig. S6**). There is notably high rhizosphere diversity specific to the Aurora location. We concluded that the detected alpha diversity varied strongly between compartments and displayed location specific patterns.

Community composition differs by location and compartment

Finally, we assessed microbial β-diversity by factors 'compartment' and 'location' using permutational multivariate analysis of variance PERMANOVA and unconstrained principle coordinate (PCo) analysis. The R^2 values of PERMANOVA were taken to approximate effect sizes of the different factors. They indicated that overall 'compartment' explained most of microbial community variation (Bacteria 25.6%, Fungi 19.7.5%), followed by 'location' (23.2%, 21.8%; **Table S3**). Unconstrained principle coordinate (PCo) analysis visualizes this conclusion with the samples largely separating these two factors along PCo axes 1 and 2, respectively (**Fig. 1**). For fungi, the pattern is similar except that the two PCo axes explained slightly less of the overall variation. This confirms that the composition of microbial communities strongly differ between compartments and different locations.

Part 2 | Compartment-wise taxonomy analysis of 'BX exudation effects'

We analyzed the soil, rhizosphere and root compartments separately for their microbiota responses to BX exudation. We analyzed the taxonomy at phylum and family levels, comparing WT and *bx1* mutant lines, as these plant lines were present at all locations. Although the phylum and family profiles were largely similar in the soil cores of WT or *bx1* plants (**Tables S6 & S7**), we found numerous bacterial and fungal phyla and families differing in abundance in the rhizosphere samples of plants that secret BXs compared to rhizosphere samples of BX mutant plants.

In the rhizosphere compartment, we found that the rhizospheres of BX-producing plants were significantly enriched in Gammaproteobacteria (WT 56%, *bx1* 36% mean relative abundance) for the field experiment in Reckenholz, but the rhizospheres of BX-deficient plants contained elevated levels of Betaproteobacteria (WT 1%, *bx1* 18%), Bacteroidetes (WT 7%, *bx1* 11%), Alphaproteobacteria (WT 6%, *bx1* 11%), Deltaproteobacteria (WT 1.2%, *bx1* 1.7%) and Glomeromycota (WT 0.1%, *bx1* 0.5%), and some unassigned fungi (WT 4%, *bx1* 11%, **Tables S6**). In Aurora we found only a few low abundant taxa being enriched in the rhizospheres of BX-deficient plants. Benzoxazinoids also influenced taxonomy at athefamily level (**Table S7)**. Among the most abundant families in BX-producing rhizosphere, Enterobacteriaceae (WT 0.99 %, *bx1* 0.46 %) were enriched in Aurora and Pseudomonadaceae (WT 63.55 %, *bx1* 41.66 %) in Reckenholz; on the contrary, Flavobacteriaceae (WT 15.54 %, *bx1* 25.56 %) in Changins and 6 families in Reckenholz, including Comamonadaceae (WT 6.65 %, *bx1* 13.35 %), Rhizobiaceae (WT 2.13 %, *bx1* 4.11 %), Cytophagaceae (WT 0.71 %, *bx1* 1.18 %) were enriched in the rhizosphere of BX-deficient plants. Some fungal families also responded to BX production, and the BX-rich rhizosphere in Changins was enriched in Ceratobasidiaceae (WT 8.68 %, *bx1* 5.90 %) and Microdiochaceae (WT 1.75 %, *bx1* 1.39 %), and Chaetomiaceae (WT 2.25 %, *bx1* 0.88 %) in Reckenholz. Glomeraceae (WT 0.13 %, *bx1* 0.54 %) were enriched in BX-deficient rhizosphere in Changins and Herpotrichiatellaceae (WT 1.04 %, *bx1* 3.46 %) in Reckenholz. Similar to the soil compartment, both bacterial and fungal alpha diversity in the rhizosphere were unaffected by BX exudation (**Table S8**). With regard to community composition, PERMANOVA quantified small yet significant effect sizes due to BX exudation of 2.2% for bacteria and 2.2% for fungi (**Fig. 2B**, **Table S9**). Slightly larger and also

significant effect sizes were found for the interaction terms of BX exudation and location, suggesting condition-specific BX effects on the microbiota.

In roots, the taxonomic analysis detected a few differences between WT and *bx1* plants (**Table S6 & S7**). At the phylum level, elevated levels of Alphaproteobacteria (WT 5.9%, *bx1* 8.8%) were found in *bx1* plants in Reckenholz. At a family level, Rhizobiaceae was enriched in BX-producing roots in Aurora (WT 11.55 %, *bx1* 10.19 %), while it was enriched in BX-depleted roots in Reckenholz (WT 3.39 %, *bx1* 5.17 %), as well as four other families including Streptomyces (WT 0.13 %, *bx1* 0.35 %) and Comamonadaceae (WT 3.25 %, *bx1* 0.5.52 %). Some fungal families were enriched in BX-producing roots, namely Mortierellaceae (WT 0.67%, *bx1* 0.64 %) and Ceratobasidiaceae (WT 17.3 %, *bx1* 6.15 %) in Changins, and Periconiaceae in Aurora (WT 3.99 %, *bx1* 1.79 %). The only fungal family enriched in BX-depleted roots was Dydimellaceae (WT 0.07%, *bx1* 0.14%), in Changins.

Part 3 | Analysis of BX-sensitive microbes

We have reported the common taxonomic patterns of BX-depleted microbes in the main text. Here we detail the location-specific enrichments and depletions of BX-sensitive microbes (**Table S14)**.

Globally, there were more BX-depleted than BX-enriched b/fOTUs, more of the BX-sensitive b/fOTUs were seen in the rhizosphere than in roots, and more bOTUs were BX-sensitive than fOTUs. On roots, diverse low-abundant bacteria were enriched on BX producing plants at all three locations, whereas a specific enrichment of abundant *Paenibacillaceae* (2 bOTUs), a *Pseudomonadaceae* and an *Enterobacteraceae* was seen only in Reckenholz. Abundant BX-enriched fOTUs were *Nectriaceae*, *Mortierellaceae, Gaeumannomyces* in Aurora and *Didymellaceae* (Reckenholz) whereas several low abundant fOTUs were found in Changins. In contrast, BX exudation depleted the following bOTUs from WT roots: abundant *Pseudomonadaceae* and *Rhizobiaceae* were depleted in Changins and several low bacteria were depleted in Aurora and Reckenholz. BX-depleted root fungi included abundant *Nectriaceae* (Changins and Reckenholz), *Herpotrichiellaceae* and several *Pleosporales* (Reckenholz) while in Aurora diverse low abundant fungi were depleted.

In the rhizospheres of BX-producing plants, mostly low-abundant bacteria and in particular *Pseudomonas* (Reckenholz) were enriched (**Table S14)**. We noticed that in Reckenholz, BX exudation enriched for diverse fOTUs, with the abundant ones belonging to *Mortierellaceae*, *Chaetomiaceae, Saccharomycetales*, and an unassigned fOTU. In Changins specifically, enriched fOTUs included several *Glomeraceae* along with an *Agaricales*. Conversely, low-abundant bacteria were depleted in rhizospheres including *Flavobacteriaceae* (Changins and Aurora), *Saprospiraceae* (Aurora and Reckenholz) and several location-specific groups (*Burkholderiales* in Changins, *Blastocatellaceae* in Aurora and *Sphingomonadaceae* and *Xanthomonadaceae* in Reckenholz). Low abundant (Aurora) or several abundant unassigned or diverse fOTUs were depleted by BX-exudation in Changins and Reckenholz, respectively.

SUPPLEMENTARY FIGURES:

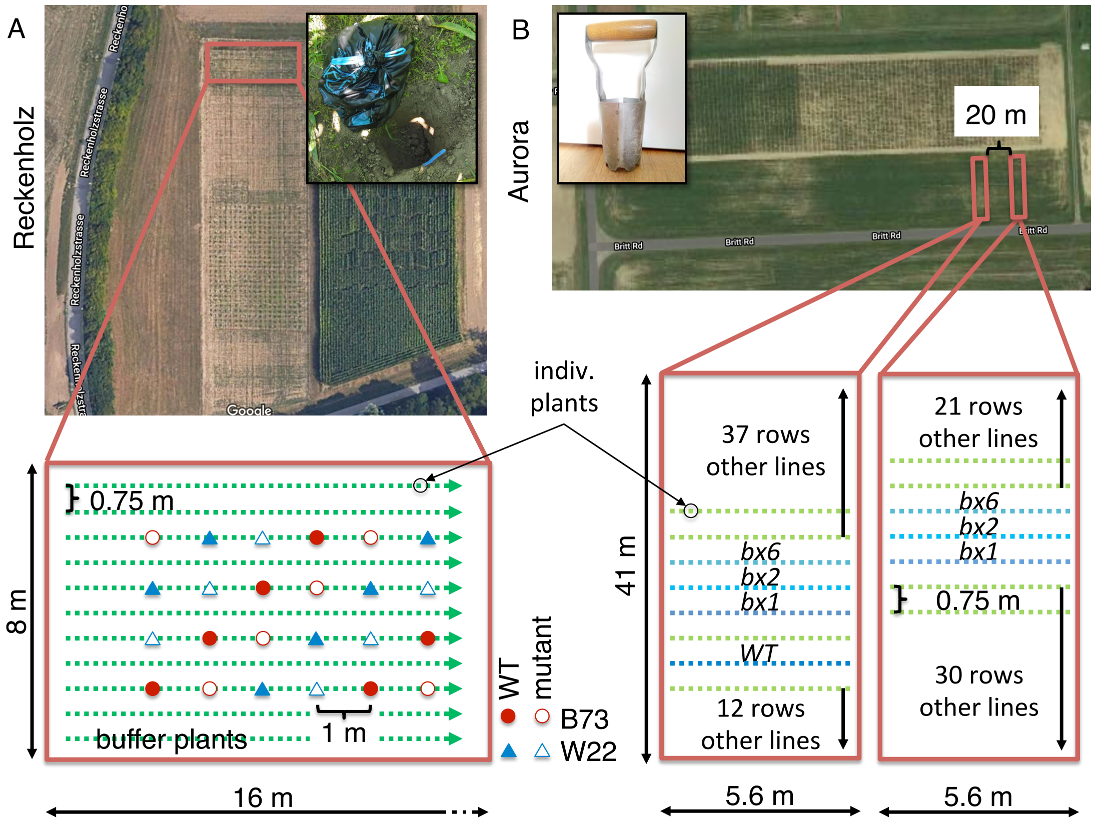

Figure S1 | Setup of field experiments

The aerial views display the fields used for the experiment in Reckenholz (**A**, Parcel 209) and Aurora (**B**, Field U). The fields can be located with the coordinates given in the methods. Red boxes frame the areas of the fields that were used and they link to a scheme of the experimental designs. In Reckenholz, the test plants were spaced by 1 m between buffer plants whereas the test plants in Aurora were sown in rows. Plant genotypes are indicated by symbols and color (A) or directly labeled (B). The inlets visualize the sampling methods to collect the soil cores (A) or the soil cylinders (B).

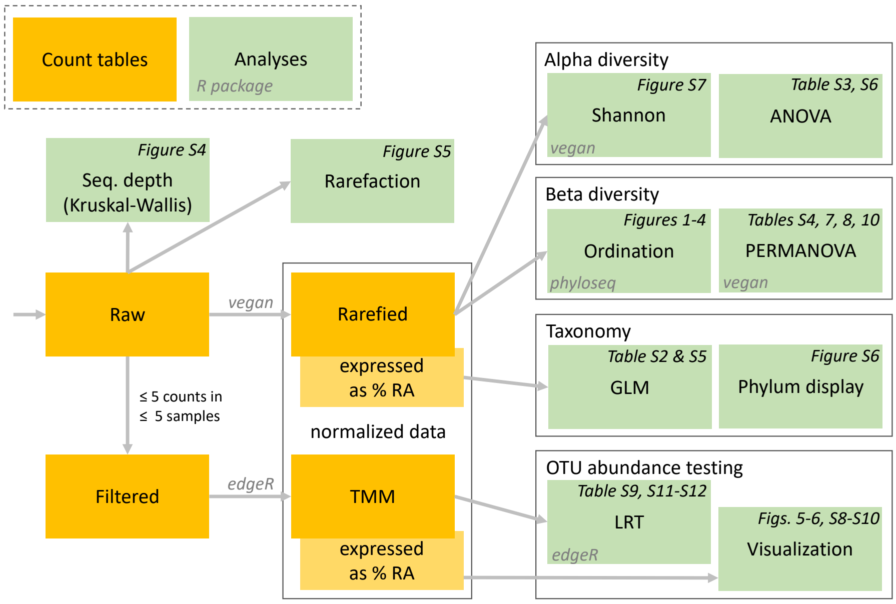

Figure S2 | Analysis steps

The schematic flow diagram illustrates the analysis steps in R. Individual types of normalization steps, analyses or statistical tests are indicated with the blue boxes. Larger grey boxes segment the analysis and indicate the major R-packages that were used in alpha- and beta diversity analyses, differential abundance testing and network analysis. Analysis outputs (Figures and Tables) are indicated in red at their respective analysis steps.

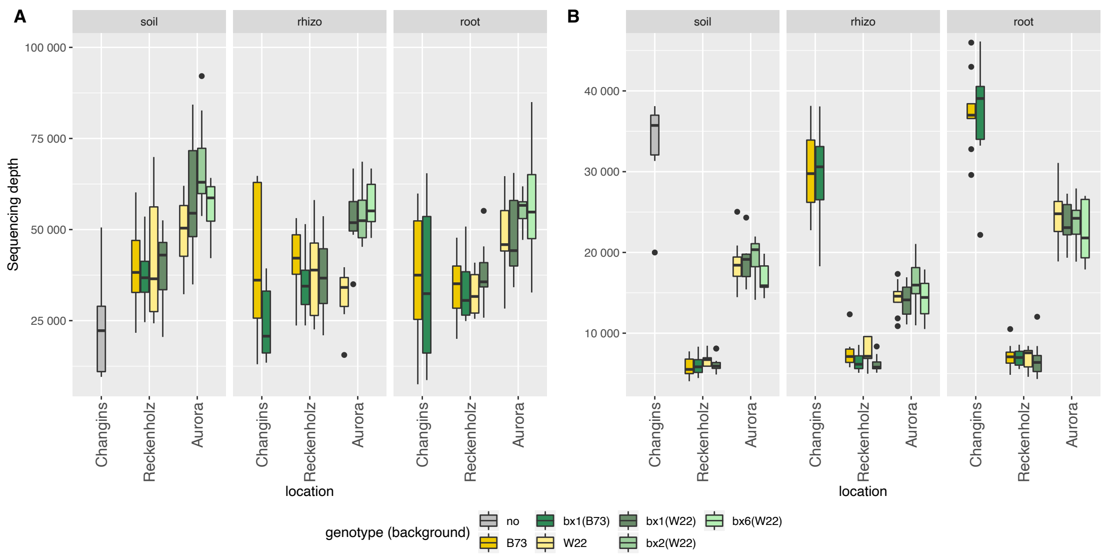

Figure S3 | Sequencing effort by sample groups

Sequencing depths of (A) bacterial and (B) fungal community profiles. Panels show different compartments, and the different genotypes for each location are shown within the panels. Mean sequencing depths differ significantly between groups of samples (Kruskal-Wallis test, $P < 0.05$, **Supplementary Data S3**).

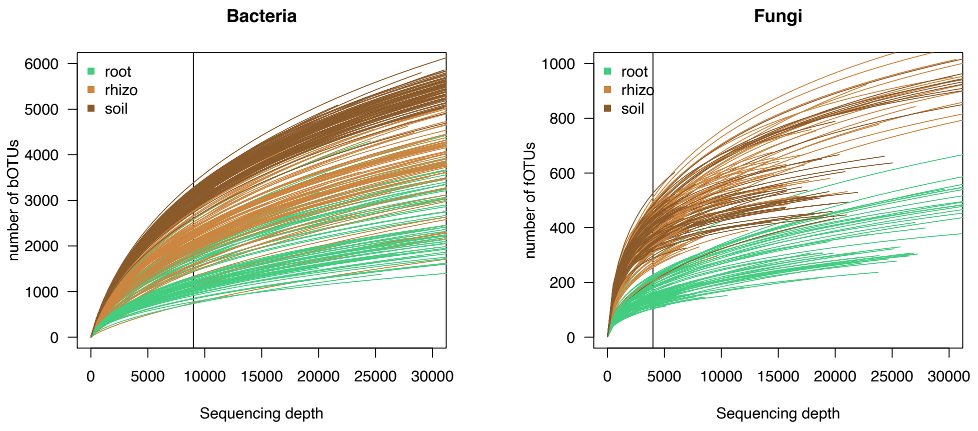

Figure S4 | Sampling intensity analysis

Rarefaction curve for (A) bacterial and (B) fungal OTU richness. Black lines indicate rarefaction thresholds (9,000 and 4,000 sequences per sample) used for alpha diversity analysis.

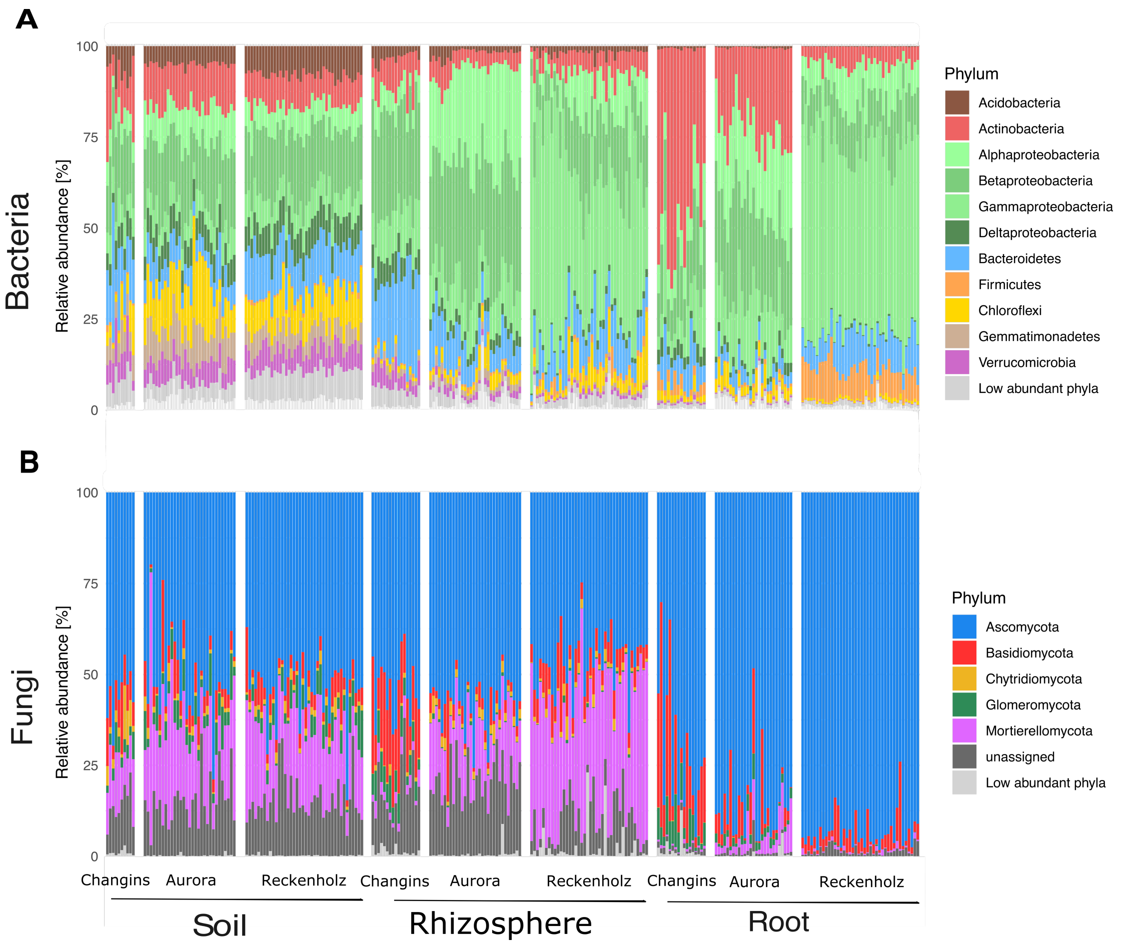

Figure S5 | Taxonomy

Taxonomy profiles of (A) bacteria (B) fungi at the class level for Proteobacteria and at the phylum level for all other phyla. Different facets in one plot show different samples (sample compartment and location), with all genotypes and replicates. Phyla are considered low abundant when they are below 1% of relative abundance. The statistical testing between the different locations and compartments is documented in **Table S3** and the analysis of BX effects in each compartment in **Table S6**.

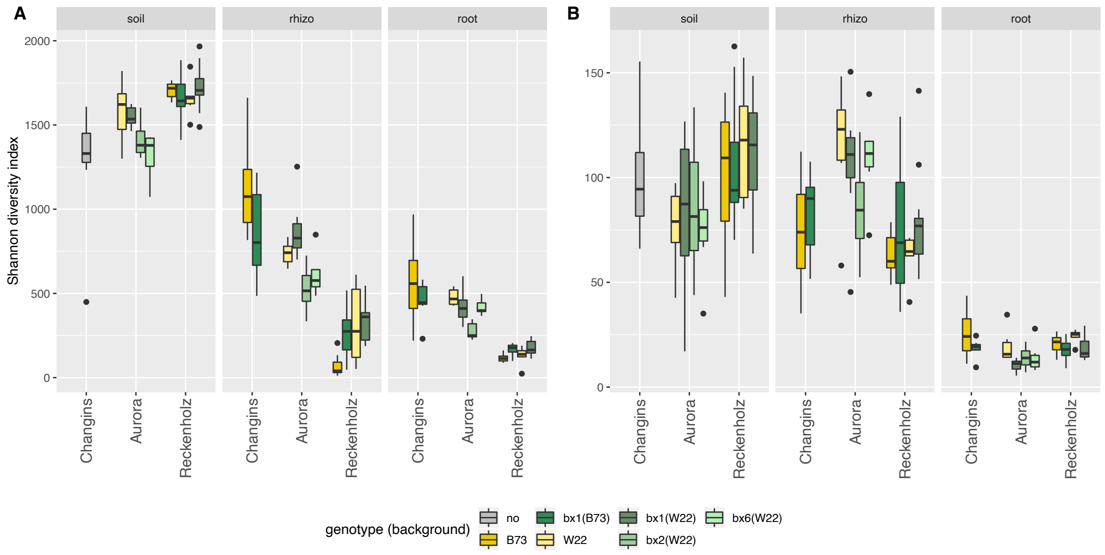

Figure S6 | Alpha diversity

Alpha diversity was measured with the Shannon index for (A) bacteria and (B) fungi on rarefied data (9,000 and 4,000 sequences per sample for bacteria and fungi, respectively), for different sample compartment (plot facets) and locations. The statistical comparisons of the different locations and compartments is documented in **Table S4**.

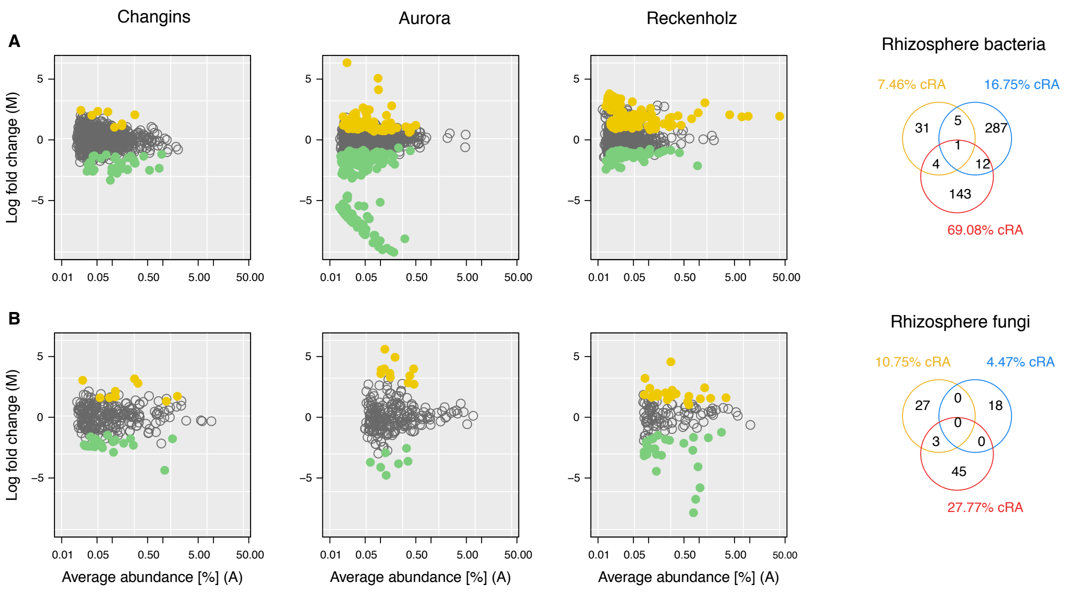

Figure S7 | BX-sensitive rhizosphere microbes across locations

The MA plots display the average abundance (in log count per million, CPM) and the log-fold change of all b/fOTUs plotted on the x- and y-axes, respectively. b/fOTUs being differentially abundant between wild-type and *bx1* mutant lines (BX-sensitive OTUs) were determined by edgeR analysis (FDR < 0.05, **Table S13**). Colors refer to enriched b/fOTUs in wild-type (yellow) or *bx1* mutant (green) lines. (A) reports the rhizosphere bacteria and (B) the rhizosphere fungi at the locations Changins (yellow), Aurora (blue) and Reckenholz (red). The comparison of BX-sensitive rhizosphere b/fOTUs between locations is visualized with the Venn diagrams. The % cRA represents the cumulated relative abundance of all detected differentially abundant b/fOTUs for each location.

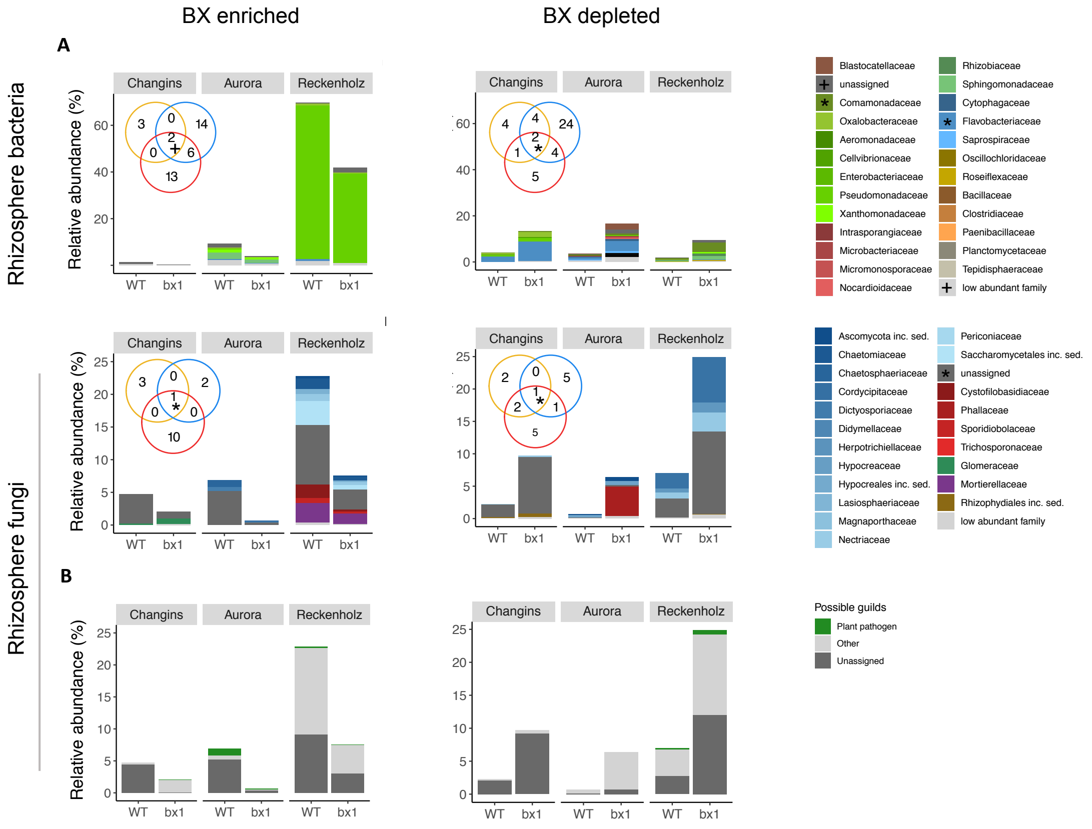

Figure S8 | Taxonomic patterns of BX-sensitive rhizosphere microbes

The bar plots depict the mean relative abundances (in %) for each location and taxonomies of all rhizosphere bOTUs (upper panels) and rhizosphere fOTUs (lower panels) that differed significantly in abundance between wild-type (WT) and *bx1* mutant lines (i.e., the BX-sensitive b/fOTUs as determined by edgeR analysis, FDR < 0.05, **Table S13**). The BX-enriched (left panels) and BX-depleted taxa (right panels) correspond to the same yellow (enriched in WT) and green (enriched in *bx1*) b/fOTUs of **Fig. S7**, respectively. Individual b/fOTUs are displayed in a stacked manner sorted by their taxonomic assignment at family level. The Venn diagram insets compare the family assignments of the BX-sensitive taxa between the locations Changins (yellow), Aurora (blue) and Reckenholz (red). Overlapping family assignments are indicated in the plot or marked in the taxonomy legend. (B) visualizes the proportion of assignments to 'plant pathogen' among the FUNGuild annotations. The sets of BX-enriched and BX-depleted rhizosphere fOTUs from each location were annotated individually to their ecological guilds.

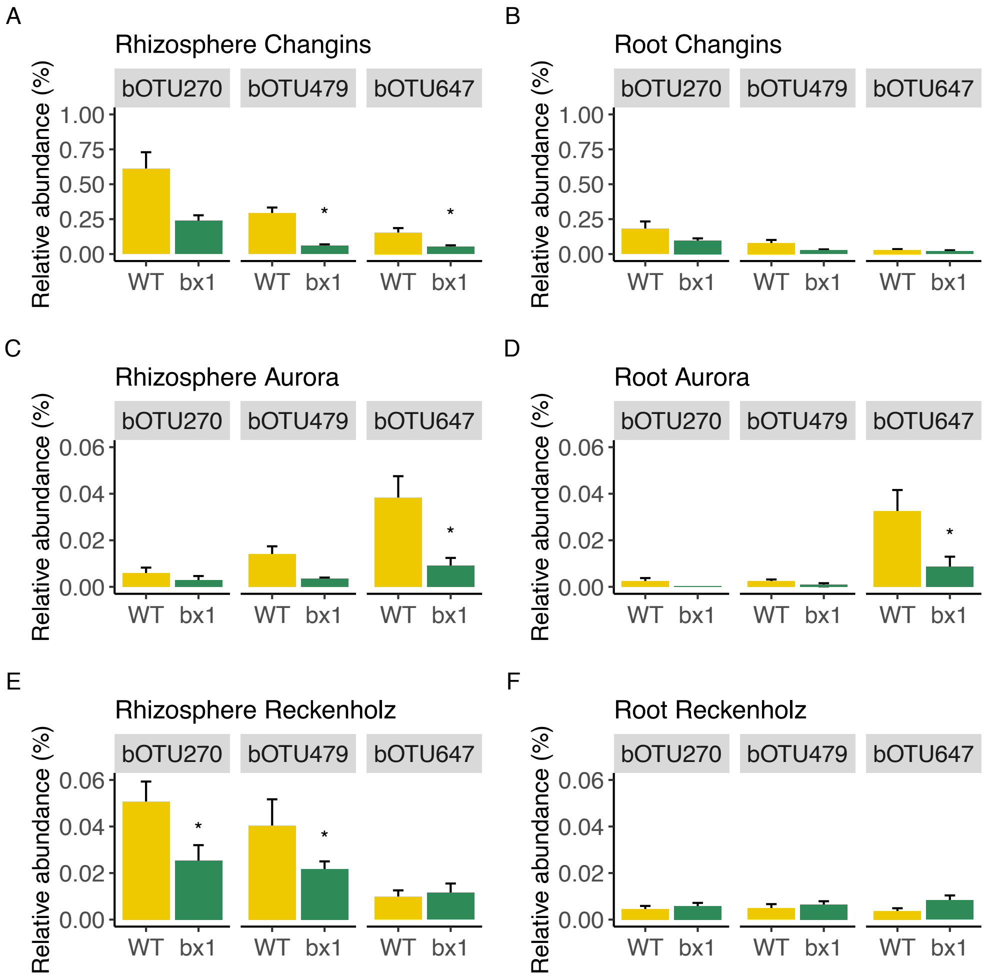

Figure S9 | Abundance of *Methylophilaceae* bOTUs across compartments and locations

Bar graphs display the mean abundance (± s.e.m.) of the *Methylophilales* bOTU479 and the two *Methylophilaceae* bOTUs #270 and #647 in rhizosphere (A,C,E) and root (B,D,F) samples of wild-type (WT) and *bx1* mutant lines at all three locations. Asterisks mark significant differences between WT and *bx1* as determined by edgeR analysis (FDR < 0.05, **Table S13**).

SUPPLEMENTARY TABLES:

Table S1 | Deposition of raw sequence data

Location	Data	MiSeq	Study accession[1]	Sample ID[1]	Publication[2]
Reckenholz	16S	Run 09	PRJEB27162	SAMEA6521205	Bodenhausen et al., 2019
Reckenholz	ITS	Run 13	PRJEB36599	SAMEA8117518	this study
Aurora	16S	Run 11	PRJEB20127	SAMEA4698767	Hu et al., 2018
Aurora	ITS	Run 12	PRJEB36599	SAMEA8117517	this study

[1]At the European Nucleotide Archive (http://www.ebi.ac.uk/ena)
[2]In our laboratory, we typically sequence several different experiments in a single MiSeq run. Therefore, it is possible that the raw data was deposited previously in the context of an earlier study.

Table S2 | Soil characteristics

Physical and chemical characteristics of the three soils from the locations Changins, Aurora and Reckenholz were determined in 1:10 water ('…-H_2O', proxy for plant available nutrients) and 1:10 acetate-ammonium EDTA ('…-EDTA', proxy for reserve nutrients) extracts. Total iron was measured in nitric acid ('Fe-HNO_3') extracts and soil texture was measured by fractionation. Letters indicate significant differences between locations (*P*-value < 0.05, Tukey HSD test).

		Changins			Aurora			Reckenholz		
		mean	SD		mean	SD		mean	SD	
P-H_2O		3.2	1.1	b	5.7	0.6	a	5.3	0.4	a
K-H_2O		44.8	5.0	a	17.4	1.9	b	23.0	1.6	b
Mg-H_2O		14.0	0.4	c	32.3	1.7	a	20.5	0.3	b
Ca-EDTA		346.7	17.9	a	113.0	1.9	b	101.1	6.7	b
N-H_2O		145.6	9.2	a	34.9	5.9	b	19.6	1.7	b
NH4-H_2O		9.2	1.6	a	10.1	1.9	a	10.6	0.4	a
Na-H_2O		8.5	0.6	a	2.8	0.3	b	4.5	3.2	ab
Fe-H_2O	mg kg^{-1}	2.6	0.3	c	14.9	1.1	a	9.9	1.3	b
Bor-H_2O		0.13	0.01	c	0.23	0.01	a	0.16	0.01	b
P-EDTA		64.6	4.4	a	54.0	3.6	b	63.2	2.8	a
K-EDTA		210.9	11.0	a	75.5	2.9	c	185.5	1.8	b
Mg-EDTA		177.0	11.7	c	415.4	10.0	b	513.0	14.2	a
Ca-EDTA		7883.7	622.5	a	2941.0	54.6	c	4601.0	153.0	b
Mn-EDTA		235.3	7.0	c	324.2	7.5	a	262.9	7.4	b
Fe-HNO_3	g kg^{-1}	23.2	1.1	a	25.1	2.4	a	20.9	1.0	b
sand	%	30.5	4.3	b	40.4	2.8	a	37.5	2.4	a
silt	%	37.1	2.1	a	37.9	1.3	a	37.8	1.6	a
clay	%	30.5	3.4	a	18.5	1.9	c	24.8	0.8	b
pH		7.8	0.4	a	7.1	0.4	b	6.9	0.4	b

Table S3 | Taxonomic analysis comparing locations and compartments

The table reports for all sample groups (location * compartment) the F statistic together with the corresponding P value (adjusted for multiple hypothesis testing, following Bonferroni Hochberg) and the relative average abundances [%]. Locations are abbreviated with Ch=Changins, Au=Aurora and Re=Reckenholz. Compartments are abbreviated with So=Soil, Rh=Rhizosphere and Ro=Root. The columns with the posthoc Tukey test results is indicated with "_T" for each sample group. Different letters are used for groups that differ significantly in their abundances (alpha=0.01).

-> See supplementary excel file (Table_S3_Phylum_statistics.xlsx)

Table S4 | Alpha diversity analysis comparing locations and compartments

Statistics of Shannon diversity between compartments (soil, rhizosphere and root) at each location (Changins, Aurora and Reckenholz; Tukey HSD test). Different letters correspond to groups that are significantly different from each other in Shannon diversity at $P < 0.05$.

Location	Compartment	Bacteria		Fungi	
Changins	Soil	1295	ef	99.44	cde
	Rhizosphere	1012	de	78.69	cd
	Root	520.6	bc	22.74	b
Aurora	Soil	1468	ef	79.43	c
	Rhizosphere	687.2	cd	104.7	de
	Root	401.5	b	14.2	a
Reckenholz	Soil	1691	f	107.5	e
	Rhizosphere	241.1	a	71.64	c
	Root	151.2	a	19.59	b

Table S5 | Beta diversity analysis comparing locations and compartments

Effects of location and compartment on beta diversity of bacterial and fungal diversity. R2 and *P*-values of PERMANOVA on Bray-Curtis distances are presented.

	**location**		**compartment**	
	R2	*P*	R2	*P*
Bacteria	0.2325	**0.001**	0.1969	**0.001**
Fungi	0.2178	**0.001**	0.2561	**0.001**

Table S6 | Phylum level taxonomic analysis comparing BX effects in each compartment

The table reports the phylum level taxonomic analysis for bacteria and fungi and for each compartment the F statistic together with the corresponding P value (adjusted for multiple hypothesis testing, following Benjamini-Hochberg. The table also displays the relative average abundances [%] per sample group (location * genotype). Locations are abbreviated with Ch=Changins, Au=Aurora and Re=Reckenholz. Genotypes are abbreviated with WT=wildtype and bx for the bx1 mutant. The columns with the posthoc Tukey test results is indicated with "_T" for each sample group. Different letters are used for groups that differ significantly in their abundances (alpha=0.01).

-> See supplementary excel file (Table_S6_Phylum_BX_statistics)

Table S7 | Family level taxonomic analysis comparing BX effects in each compartment

The table reports the family level taxonomic analysis for bacteria and fungi and for each compartment the F statistic together with the corresponding P value (adjusted for multiple hypothesis testing, following Benjamini-Hochberg). The table also displays the relative average abundances [%] per sample group (location * genotype). Locations are abbreviated with Ch=Changins, Au=Aurora and Re=Reckenholz. Genotypes are abbreviated with WT=wildtype and bx for the bx1 mutant. The columns with the posthoc Tukey test results is indicated with "_T" for each sample group. Different letters are used for groups that differ significantly in their abundances (alpha=0.01).

-> See supplementary excel file (Table_S7_Family_BX_statistics.xlsx)

Table S8 | Alpha diversity analysis comparing BX effects in each compartment

Effect of BX production (Wild type vs. mutant) on Shannon diversity within compartments. *F* and *P*-values (if significant, then in bold) from ANOVA are presented.

	**Root**		**Rhizosphere**		**Soil**	
	F	*P*	F	*P*	F	*P*
Bacteria	0.0087	0.9219	1.882	0.1744	0.0028	0.9579
Fungi	**14.11**	**0.0003**	0.5741	0.451	0.1515	0.6987

Table S9 | Beta diversity analysis comparing BX and location effects in each compartment

Location (L) and genotype (G) effects of BX-exudation on beta diversity of bacterial and fungal communities within compartments. R2 and *P*-values of PERMANOVA on Bray-Curtis distance are presented. Significant effects are in bold.

		**Soil**		**Rhizosphere**		**Root**	
		R2	*P*	R2	*P*	R2	*P*
Bacteria	location	**0.3376**	**0.001**	**0.511**	**0.001**	**0.5349**	**0.001**
	genotype	0.01344	0.245	**0.02244**	**0.007**	**0.01643**	**0.048**
	L * G	0.01383	0.221	**0.03635**	**0.003**	**0.02155**	**0.097**
Fungi	location	**0.2612**	**0.001**	**0.4698**	**0.001**	**0.422**	**0.001**
	genotype	0.01472	0.263	**0.02137**	**0.009**	**0.06145**	**0.001**
	L * G	0.02111	0.076	**0.02499**	**0.049**	**0.04046**	**0.002**

Table S10 | Beta diversity analysis comparing genetic background and genotype effects in each compartment (Reckenholz experiment)

Factorial (A) PERMANOVA analysis and (B) pairwise PERMANOVA analysis of bacterial and fungal communities at the Reckenholz location testing for effects of compartment (soil, root and rhizosphere), genetic background (B73, W22) and plant genotype (WT, *bx1*). R2 and *P*-values of PERMANOVA using the model '*~compartment* + *background* + *genotype*' on Bray-Curtis distance are presented. For pairwise PERMANOVA, R2 values are reported and significant (FDR < 0.05) effects marked in bold.

(A)	**Compartment**		**Background**		**Genotype**	
	R2	*P*	R2	*P*	R2	*P*
Bacteria	**0.3486**	**0.001**	**0.02734**	**0.004**	**0.03431**	**0.002**
Fungi	**0.4987**	**0.001**	**0.02524**	**0.005**	**0.04461**	**0.001**

(B)		***bx1*(b73) - *bx1*(W22)**	***bx1*(b73) - B73**	***bx1*(b73) - W22**	***bx1*(W22) - B73**	***bx1*(W22) - W22**	**B73 - W22**
Bacteria	Root	0.07705	0.0988	0.1365	0.1266	0.08215	0.1203
Fungi		0.1658	0.2182	0.2131	**0.3666**	0.2743	0.1203
Bacteria	Rhizo.	0.07349	0.2425	0.05906	**0.3524**	0.08487	0.2326
Fungi		0.1096	0.1701	0.1374	**0.2402**	0.1576	0.2042

Table S11 | zOTUs differing by genetic background or genotype (Reckenholz experiment)

The supplementary table lists, for bacteria and fungi of the root and the rhizosphere, all zOTUs differing significantly between genetic backgrounds (B73, W22) or genotypes (WT, *bx1*) based on edgeR analysis. The table lists the zOTU-ID, taxonomy, the mean log abundance in counts per million [logCPM] and log fold change [logFC] between genetic backgrounds or genotypes and the statistic of the likelihood ratio test [LR], its *P*-value [PValue], its FDR-corrected *P*-value [FDR] and the proportion of total sequence abundance [percAllSeq].

-> See supplementary excel file (Table_S11_Reckenholz_OTU_statistics.xlsx)

Table S12 | Beta diversity analysis comparing mutant genotypes in each compartment (Aurora experiment)

Factorial (A) PERMANOVA analysis and (B) pairwise PERMANOVA analysis of bacterial and fungal communities at the Aurora location testing for effects of compartment (soil, root and rhizosphere), plant genotypes (WT, *bx1*, *bx2* and *bx6*) and interaction between these two terms. R2 and *P*-values of PERMANOVA using the model '*~compartment * genotype*' on Bray-Curtis distance are presented. For pairwise PERMANOVA R2 values are reported and significant (FDR < 0.05) effects marked in bold.

(A)	**Compartment**		**Genotype**		**Comp. * Gen.**	
	R2	*P*	R2	*P*	R2	*P*
Bacteria	**0.3686**	**0.001**	**0.08179**	**0.001**	**0.04973**	**0.035**
Fungi	**0.4049**	**0.001**	**0.06425**	**0.023**	0.02467	0.644

(B)		***bx1 – bx2***	***bx1 – bx6***	***bx1* – WT**	***bx2 – bx6***	***bx2* – WT**	***bx6* – WT**
Bacteria	Root	**0.1405**	**0.1383**	**0.1221**	**0.1289**	**0.196**	**0.1524**
Fungi	Root	0.09296	0.1062	**0.2069**	0.07716	0.1677	0.1223
Bacteria	Rhizo.	**0.1355**	**0.2009**	**0.0875**	0.337	**0.08852**	**0.2543**
Fungi	Rhizo.	**0.1146**	0.0921	0.0875	0.0839	**0.1012**	0.0896

Table S13 | zOTUs differing by mutant genotypes (Aurora experiment)

The supplementary table lists, for bacteria and fungi of the root and the rhizosphere, all zOTUs differing significantly between among WT and the mutant lines based on edgeR analysis. The table lists the zOTU-ID, taxonomy, the mean log abundance in counts per million [logCPM] and log fold change [logFC] between WT and *bx1* and the statistic of the likelihood ratio test [LR], its *P*-value [PValue] and its FDR-corrected *P*-value [FDR] and the proportion of total sequence abundance [percAllSeq].

-> See supplementary excel file (Table_S13_Aurora_OTU_statistics.xlsx)

Table S14 | BX-sensitive zOTUs across all locations

The supplementary excel table lists for bacteria and fungi of the root and the rhizosphere all zOTUs differing significantly between WT and *bx1* mutant lines for each location based on edgeR analysis. That table lists the location, the zOTU-ID, taxonomy, the mean log abundance in counts per million [logCPM] and log fold change [logFC] between WT and *bx1* and the statistic of the likelihood ratio test [LR], its *P*-value [PValue] and its FDR-corrected *P*-value [FDR] and the proportion of total sequence abundance [percAllSeq].

-> See supplementary excel file (Table_S14_BX_OTU_statistics.xlsx)
